# Regulation of the Mitochondrion-Fatty Acid Axis for the Metabolic Reprogramming of Chlamydia trachomatis during Treatment with β-Lactam Antimicrobials

**DOI:** 10.1128/mBio.00023-21

**Published:** 2021-03-30

**Authors:** Kensuke Shima, Inga Kaufhold, Thomas Eder, Nadja Käding, Nis Schmidt, Iretiolu M. Ogunsulire, René Deenen, Karl Köhrer, Dirk Friedrich, Sophie E. Isay, Florian Grebien, Matthias Klinger, Barbara C. Richer, Ulrich L. Günther, George S. Deepe, Thomas Rattei, Jan Rupp

**Affiliations:** a Department of Infectious Diseases and Microbiology, University of Lübeck, Lübeck, Germany; b Division of Computational Systems Biology, University Vienna, Vienna, Austria; c Institute for Medical Biochemistry, University of Veterinary Medicine Vienna, Vienna, Austria; d Biological and Medical Research Center (BMFZ), Heinrich Heine University Düsseldorf, Düsseldorf, Germany; e Institute of Anatomy, University of Lübeck, Lübeck, Germany; f Institute of Chemistry and Metabolomics, University of Lübeck, Lübeck, Germany; g Division of Infectious Diseases, College of Medicine, University of Cincinnati, Cincinnati, Ohio, USA; h German Center for Infection Research (DZIF), Partner Site Hamburg-Lübeck-Borstel-Riems, Lübeck, Germany; University of Nebraska Medical Center

**Keywords:** *Chlamydia trachomatis*, citrate, STAT3, beta-lactams, fatty acids, metabolism, mitochondria, penicillin, persistence, protein tyrosine phosphatase

## Abstract

Infection with the obligate intracellular bacterium Chlamydia trachomatis is the most common bacterial sexually transmitted disease worldwide. Since no vaccine is available to date, antimicrobial therapy is the only alternative in C. trachomatis infection. However, changes in chlamydial replicative activity and the occurrence of chlamydial persistence caused by diverse stimuli have been proven to impair treatment effectiveness. Here, we report the mechanism for C. trachomatis regulating host signaling processes and mitochondrial function, which can be used for chlamydial metabolic reprogramming during treatment with β-lactam antimicrobials. Activation of signal transducer and activator of transcription 3 (STAT3) is a well-known host response in various bacterial and viral infections. In C. trachomatis infection, inactivation of STAT3 by host protein tyrosine phosphatases increased mitochondrial respiration in both the absence and presence of β-lactam antimicrobials. However, during treatment with β-lactam antimicrobials, C. trachomatis increased the production of citrate as well as the activity of host ATP-citrate lyase involved in fatty acid synthesis. Concomitantly, chlamydial metabolism switched from the tricarboxylic acid cycle to fatty acid synthesis. This metabolic switch was a unique response in treatment with β-lactam antimicrobials and was not observed in gamma interferon (IFN-γ)-induced persistent infection. Inhibition of fatty acid synthesis was able to attenuate β-lactam-induced chlamydial persistence. Our findings highlight the importance of the mitochondrion-fatty acid interplay for the metabolic reprogramming of C. trachomatis during treatment with β-lactam antimicrobials.

## Abstract (precis)

The mitochondrion generates most of the ATP in eukaryotic cells, and its activity is used for controlling the intracellular growth of Chlamydia trachomatis. Furthermore, mitochondrial activity is tightly connected to host fatty acid synthesis that is indispensable for chlamydial membrane biogenesis.

## INTRODUCTION

Each year, more than 130 million people are infected with the obligate intracellular bacterium Chlamydia trachomatis worldwide (1). Infections induce inflammatory tissue damage in the upper genital tract that is linked to severe sequelae such as pelvic inflammatory disease (PID) and infertility in women (1).

Productive C. trachomatis exhibits a unique biphasic life cycle that alternates between two morphological forms, infectious elementary bodies (EBs) and replicating reticulate bodies (RBs) (2). However, the chlamydial persistent form containing aberrant RBs is induced by several stimuli such as β-lactams or gamma interferon (IFN-γ) (3–10). While penicillin is known for one of the best-characterized *in vitro* chlamydial persistence models (3, 7, 11–13), amoxicillin is clinically considered an alternative therapeutic drug for C. trachomatis in pregnant women (14, 15). Although amoxicillin is not a front-line antimicrobial against C. trachomatis infection, β-lactams are commonly prescribed for various bacterial infections in the United States (16) and for the treatment of different bacterial sexually transmitted diseases (STDs) (3–7, 14). Acute infection with C. trachomatis is often asymptomatic (15). Therefore, amoxicillin that may be used to treat a urinary tract infection, for example, might induce persistence in patients who are asymptomatically infected with C. trachomatis (7). Importantly, it has been reported that the efficacy of first-line antimicrobials such as doxycycline and azithromycin against the persistent phenotype of C. trachomatis is reduced (3, 17). β-Lactams have also been used to uncover chlamydial basic medical science such as the mechanism of cell division (18–20).

Although C. trachomatis is able to generate its own ATP, mitochondrial activity and various mitochondrion-derived metabolites are essentially needed for its intracellular development (2, 10, 21). Genome sequencing revealed that C. trachomatis lacks many metabolic genes (22), including several genes in the chlamydial tricarboxylic acid (TCA) cycle (23). Therefore, C. trachomatis has to hijack host mitochondrion-derived 2-oxoglutarate and glutamine that is further catabolized to glutamate and 2-oxoglutarate in order to initiate the chlamydial TCA cycle (22–24).

Mitochondrion-derived citrate is an essential metabolite for the synthesis of host fatty acids (25). It is also known that C. trachomatis depends on host-derived lipids for its life cycle (26–31). Although C. trachomatis carries genes for fatty acid and chlamydial membrane synthesis, the pathogen lacks some genes that play a key role in the synthesis of unsaturated fatty acids (22, 26–28). Consequently, C. trachomatis utilizes host-derived fatty acids and lipids for its own membrane biogenesis to compensate for the truncated chlamydial lipid metabolic pathway (26–28, 32).

One of the regulators of mitochondrial activity is signal transducer and activator of transcription 3 (STAT3) (33–37). Tyrosine phosphorylation of STAT3 at position 705 (STAT3 pY^705^) is important to form active STAT3 that downregulates mitochondrial activity in primary mouse embryonic fibroblasts (34–36). Active STAT3 not only reduces mitochondrial membrane potential but also diminishes protein levels of representative components of the electron transport chain (ETC), especially complexes IV and V (34, 36). Hence, this downregulation further leads to reductions in respiratory chain activity and mitochondrial ATP production (34, 36). Furthermore, it is known that serine phosphorylation of STAT3 at position 727 (STAT3 pS^727^) localizes in mitochondria and enhances mitochondrial functions (37). While STAT3 is phosphorylated at position 705 during various cytokine receptor signaling events, including interleukin-6 (IL-6), it is negatively regulated by seven different phosphotyrosine phosphatases (PTPs) (38). Furthermore, negative regulation occurs by suppressor of cytokine signaling 3 (SOCS3) and protein inhibitor of activated STAT (PIAS) (39).

Although host mitochondrial activity is crucial for the life cycle of C. trachomatis, little is known about how C. trachomatis subverts host signaling processes and utilizes mitochondrial functions in chlamydial persistence.

In this study, we show a unique response that chlamydial metabolism switched from the TCA cycle to fatty acid synthesis in β-lactam-induced chlamydial persistence, which is linked to PTP-STAT3-regulated mitochondrial activity.

## RESULTS

### Mitochondrial activity is enhanced in productive C. trachomatis infection.

**FIG 1:**
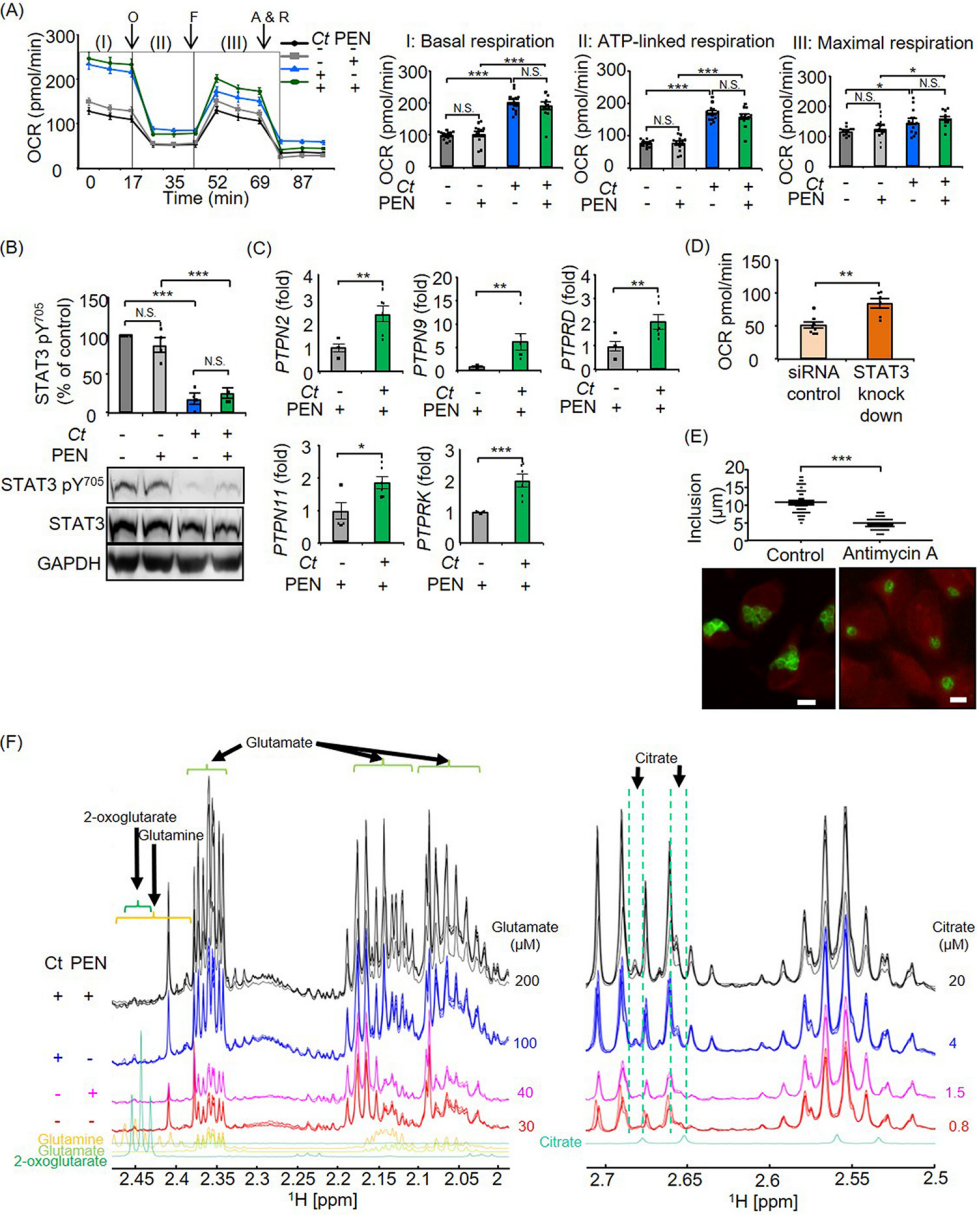
Analysis of mitochondrial activities and STAT3 regulation in productive and penicillin (PEN)-induced chlamydial persistence. (A) Mitochondrial activity was measured by using a Mito stress test kit at 24 hpi. OCR, oxygen consumption rate; O, oligomycin; F, carbonyl cyanide-4-(trifluoromethoxy)phenylhydrazone (FCCP); A & R, antimycin A plus rotenone. I, II, and III indicate basal respiration, ATP-linked respiration, and maximal respiration, respectively. (B) STAT3 pY^705^ in productive and penicillin-induced chlamydial persistence was analyzed by Western blot and densitometric analyses at 24 hpi. STAT3 pY^705^ was normalized to total STAT3 proteins. (C) Expression of PTPs in penicillin-induced chlamydial persistence was analyzed by qRT-PCR at 24 hpi. (D) Maximal respiration of siRNA control cells and STAT3 knockdown cells (STAT3HSS186130) in penicillin-induced chlamydial persistence. (E) Size of inclusions and representative images of penicillin-induced chlamydial persistence in 0.2 μM antimycin A-treated and nontreated cells at 24 hpi. Bars = 10 μm. (F) Section of ^1^H-NMR spectra expanded between 2 and 2.45 ppm and between 2.5 and 2.7 ppm of deproteinized extracts of control cells (red lines), penicillin-treated cells (magenta lines), C. trachomatis (*Ct*)-infected cells (blue lines), and C. trachomatis-infected cells in the presence of penicillin (black lines). Signals arising from each of these metabolites are marked. Deconvolution of overlapping signals was achieved using Chenomx software. Glutamate and citrate concentrations are shown on the right side of each line (*n* = 12 to 14 from four independent experiments [A], *n *= 4 [B], *n *= 6 [C], *n *= 6 to 8 from two independent experiments [D], *n* = 57 to 77 [E], and *n *= 3 to 5 [F]) (means ± SEM) (*, *P ≤ *0.05; **, *P ≤ *0.01; ***, *P ≤ *0.001; N.S., not significant [by Sidak’s multiple-comparison test {A and B} and Student’s *t* test {C to E}]).

To investigate mitochondrial responses in C. trachomatis infection, we first measured mitochondrial respiration in the absence of antimicrobials. Mitochondrial activity as defined by basal respiration, ATP-linked respiration, and maximal respiration was increased in C. trachomatis-infected cells compared to noninfected control cells at 24 h postinfection (hpi) (Fig. 1A). In accordance with the upregulation of mitochondrial activity, the complex IV and V proteins MT-CO1 (mitochondrially encoded cytochrome oxidase I) and ATP5A1 (ATP synthase, H^+^ transporting, mitochondrial F1 complex, alpha subunit 1, cardiac muscle) in the ETC were upregulated in C. trachomatis*-*infected cells at 24 hpi (see Fig. S1A and B in the supplemental material). Furthermore, chlamydial growth was inhibited when cells were treated with a mitochondrial ETC complex III inhibitor, antimycin A (Fig. S1C).

10.1128/mBio.00023-21.1 FIG S1 Impact of productive C. trachomatis (*Ct*) infection on mitochondrial activities and STAT3 regulation. (A and B) Protein amounts of MT-CO1 (mitochondrially encoded cytochrome oxidase I) (A) and ATP5A1 (ATP synthase, H^+^ transporting, mitochondrial F1 complex, alpha subunit 1, cardiac muscle) (B) in productive C. trachomatis infection were analyzed by Western blot and densitometric analyses at 24 hpi. MT-CO1 and ATP5A1 were normalized to GAPDH. (C) Size of inclusions and representative images of productive chlamydial infection in 0.2 μM antimycin A-treated and nontreated cells at 24 hpi. Bar = 10 μm. (D and E) STAT3 pS^727^ (D) and SOCS3 (E) protein amounts in productive C. trachomatis infection were analyzed by Western blot and densitometric analyses at 24 hpi. STAT3 pS^727^ was normalized to total STAT3. SOCS3 was normalized to GAPDH. (F) Expression of PTPs in productive C. trachomatis infection was analyzed by qRT-PCR at 24 hpi. (G) Effect of Na_3_VO_4_ on STAT3 pY^705^ in productive C. trachomatis infection. STAT3 pY^705^ was normalized to total STAT3. (H and I) Western blot analysis showed that STAT3 was silenced by transfection of specific siRNAs, STAT3HSS186130 and STAT3HSS186131, within 72 h. (J and K) Representative Western blot images of total amounts of phosphorylated STAT3 reduced by transfection of specific siRNAs, STAT3HSS186130 and STAT3HSS186131, at 72 h. (L and M) C. trachomatis infection rates between siRNA control cells and STAT3 knockdown cells (STAT3HSS186130 and STAT3HSS186131). (N and O) Maximal respiration of siRNA control cells and STAT3 knockdown cells (STAT3HSS186130 and STAT3HSS186131) in productive infection. (P and Q) Recoverable C. trachomatis in siRNA control cells and STAT3 knockdown cells (STAT3HSS186130 and STAT3HSS186131). (R) STAT3 pY^705^ in C. trachomatis-infected primary mouse fibroblast cells was analyzed by Western blot and densitometric analyses at 24 hpi. STAT3 pY^705^ was normalized to total STAT3. (S) Representative images of C. trachomatis infection in primary mouse fibroblast cells at 24 hpi. Green, chlamydial inclusions; red, Evans blue counterstaining. Bar = 10 μm. (T) Expression of PTPs in C. trachomatis-infected primary mouse fibroblast cells was analyzed by qRT-PCR at 24 hpi. OCR, oxygen consumption rate (*n *= 7 [A and B], *n *= 30 to 44 [C], *n *= 16 [D], *n *= 7 [E], *n *= 5 [F], *n *= 6 [G], *n *= 3 [H and I], *n *= 2 [J and K], *n *= 4 [L and M], *n *= 9 from four independent experiments [N], *n *= 11 to 15 from three independent experiments [O], *n *= 4 [P and Q], and *n *= 4 to 5 [R and T]) (means ± SEM) (*, *P ≤ *0.05; **, *P ≤ *0.01; ***, *P ≤ *0.001; N.S., not significant [by Student’s *t* test {A to F, L to R, and T} and Sidak’s multiple-comparison test {G to I}]). Download FIG S1, TIF file, 1.2 MB. Copyright © 2021 Shima et al. 2021 Shima et al. https://creativecommons.org/licenses/by/4.0/ This content is distributed under the terms of the Creative Commons Attribution 4.0 International license.

Mitochondrial activity is both positively and negatively regulated by STAT3 (33, 34, 37). However, nothing is known about this regulation in C. trachomatis infection as of now. Therefore, we analyzed the phosphorylation level of STAT3. C. trachomatis infection strongly reduced STAT3 pY^705^ but not STAT3 pS^727^ at 24 hpi (Fig. 1B and Fig. S1D). To gain insight into the mechanism of reduced STAT3 pY^705^, we investigated the expression levels of SOCS3 and PTPs. Although STAT3 pY^705^ was reduced in C. trachomatis-infected cells at 24 hpi, there was no difference in SOCS3 protein amounts between C. trachomatis-infected cells and noninfected control cells (Fig. S1E). On the other hand, PTPN2, PTPN9, and PTPRK were significantly upregulated in C. trachomatis-infected cells compared to noninfected cells at 24 hpi (Fig. S1F). By inhibiting the PTPs using sodium orthovanadate, the effect of C. trachomatis infection on STAT3 pY^705^ expression was blocked (Fig. S1G).

To elucidate the role of STAT3 phosphorylation in host mitochondrial activity as well as chlamydial replication, we silenced STAT3 using RNA interference (RNAi) (small interfering RNA [siRNA]) (Fig. S1H and J). Inhibition of STAT3 had no impact on the overall infection rates of C. trachomatis (Fig. S1L). However, mitochondrial respiration was increased following knockdown of STAT3 in C. trachomatis-infected cells at 24 hpi compared to control cells (Fig. S1N). Concomitant with this finding, infectious progeny was increased following the knockdown of STAT3 (Fig. S1P). Similar results were obtained using another siRNA targeting a different sequence of STAT3 (Fig. S1I, K, M, O, and Q). These results indicate that STAT3 functions as a regulator of mitochondrial activity in C. trachomatis infection, thereby controlling infectious progeny of C. trachomatis.

### Regulation of STAT3 and mitochondrial activity in C. trachomatis-infected cells during treatment with β-lactam antimicrobials.

We next assessed whether the observed regulation of PTPs-STAT3 and mitochondrial respiration was altered during treatment with β-lactam antimicrobials. Transmission electron microscopy (TEM) and/or immunofluorescence staining revealed chlamydial persistent morphology indicated by aberrant RBs in penicillin or a therapeutically relevant serum concentration (7) of amoxicillin (Fig. S2A to C). Aberrant RBs are known to be in a viable but noncultivable state, and they can reenter the productive developmental cycle when β-lactam antimicrobials are removed (7). Accordingly, a decrease in infectious progeny was observed in penicillin- or amoxicillin-treated cells, and aberrant RBs were reactivated by the removal of β-lactam antimicrobials (Fig. S2D to F).

10.1128/mBio.00023-21.2 FIG S2 Analysis of the morphology, recovery, and reactivation of β-lactam-induced chlamydial persistence. (A) The morphology of penicillin (PEN)-induced chlamydial persistence was analyzed by TEM. Bar = 10 μm. (B) Size of aberrant RBs in penicillin treatment. (C) The morphology of β-lactam-induced chlamydial persistence was analyzed by immunofluorescence staining. Immunofluorescence staining was performed with mouse anti-chlamydial LOS antibody to visualize chlamydial inclusions (green). Evans blue counterstaining of host cells is in red. Bar = 10 μm. (D) Experimental setting for the analysis of recovery and reactivation of β-lactam-induced persistent C. trachomatis infection. C. trachomatis-infected cells were treated with or without 1 U/ml penicillin or 11 μg/ml amoxicillin (AMX) at the time of infection to induce chlamydial persistence. C. trachomatis was cultured for 24 h. For the recovery assay, C. trachomatis was further cultured for 48 h without medium change. For reactivation of persistence, medium was changed to fresh medium in the absence of antimicrobials, and cells were further incubated for another 48 h. +, presence; −, absence. (E) Recovery and reactivation assays in penicillin-induced persistent infection. (F) Recovery and reactivation assays in amoxicillin-induced persistent infection. (*n *= 7 to 10 [B], *n *= 3 [E], and *n *= 3 to 6 [F]) (means ± SEM) (**, *P ≤ *0.01; ***, *P ≤ *0.001 [by Student’s *t* test]). Download FIG S2, TIF file, 2.1 MB. Copyright © 2021 Shima et al. 2021 Shima et al. https://creativecommons.org/licenses/by/4.0/ This content is distributed under the terms of the Creative Commons Attribution 4.0 International license.

During penicillin treatment, basal respiration, ATP-linked respiration, and maximal respiration were elevated, as seen in productive infection at 24 hpi (Fig. 1A). In line with this result, STAT3 pY^705^ was reduced, similar to the observation in productive infection at 24 hpi (Fig. 1B). Concordant with the downregulation of STAT3 pY^705^, not only PTPN2, PTPN9, and PTPRK but also PTPN11 and PTPRD were upregulated by penicillin treatment at 24 hpi (Fig. 1C). To elucidate the link between reduced STAT3 pY^705^ and enhanced mitochondrial activity in penicillin-induced chlamydial persistence, we inhibited STAT3 using siRNA (Fig. S1H and J). In penicillin-induced chlamydial persistence, mitochondrial respiration was increased in STAT3 knockdown cells compared to control cells at 24 hpi (Fig. 1D). Accordingly, the formation of enlarged RBs and chlamydial inclusions was inhibited when the mitochondrial ETC was blocked by antimycin A at 24 hpi (Fig. 1E).

Since C. trachomatis needs mitochondrion-derived metabolites for its intracellular survival, we performed a targeted analysis of the 2-oxoglutarate family, including glutamine, glutamate, and 2-oxoglutarate, as well as citrate using nuclear magnetic resonance (NMR) spectroscopy. Each of these metabolites can be detected in NMR spectra of cell extracts by simulating the signals of each spin system using Chenomx software. This software also allows one to calculate the concentration of the metabolite based on signal intensities compared to the deuterated TMSP [3-(trimethylsilyl)-2,2,3,3-tetradeuteropropionic acid] reference. This analysis revealed that glutamate, which can be catabolized to 2-oxoglutarate, was increased in C. trachomatis infection compared to control cells, and this elevation was not suppressed by penicillin treatment at 24 hpi (Fig. 1F). Moreover, we detected a 5-fold higher concentration of citrate in penicillin-induced persistent infection than in productive infection (Fig. 1F).

We showed a similar trend of PTP-STAT3 regulation and mitochondrial activation in amoxicillin treatment at 24 hpi (Fig. S3A to C), indicating that mitochondrial activity plays a pivotal role not only in productive infection but also in penicillin- and amoxicillin-induced chlamydial persistence.

10.1128/mBio.00023-21.3 FIG S3 Analysis of mitochondrial activities and STAT3 regulation in amoxicillin (AMX)-induced chlamydial persistence. (A) STAT3 pY^705^ in chlamydial persistence was analyzed by Western blot and densitometric analyses at 24 hpi. STAT3 pY^705^ was normalized to total STAT3 proteins. (B) Expression of PTPs was analyzed by qRT-PCR at 24 hpi. (C) Mitochondrial activity was measured by a Mito stress test kit at 24 hpi. OCR, oxygen consumption rate; O, oligomycin; F, FCCP; A&R, antimycin A plus rotenone. I, II, and III indicate basal respiration, ATP-linked respiration, and maximal respiration, respectively. *Ct*, C. trachomatis (*n *= 5 [A], *n *= 5 [B], and *n *= 8 to 11 from three independent experiments [C]) (means ± SEM) (**, *P ≤ *0.01; ***, *P ≤ *0.001; N.S., not significant [by Sidak’s multiple-comparison test {A} and Student’s *t* test {B and C}]). Download FIG S3, TIF file, 1.4 MB. Copyright © 2021 Shima et al. 2021 Shima et al. https://creativecommons.org/licenses/by/4.0/ This content is distributed under the terms of the Creative Commons Attribution 4.0 International license.

### Fluorescence lifetime imaging microscopy of reduced NAD(P)H in productive C. trachomatis and penicillin-induced chlamydial persistence.

**FIG 2:**
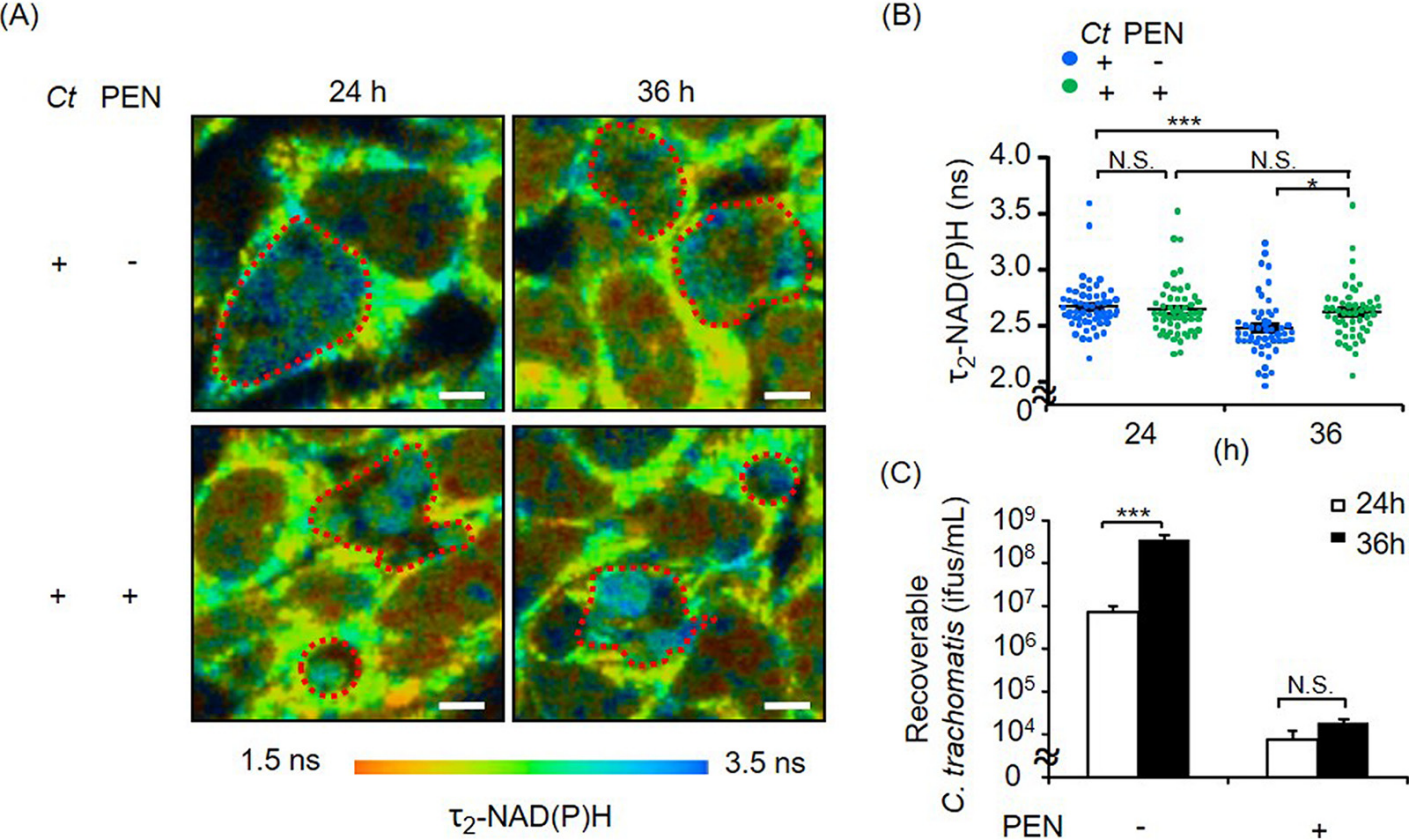
FLIM of NAD(P)H in productive C. trachomatis (*Ct*) and penicillin (PEN)-induced chlamydial persistence. τ_2_-NAD(P)H in chlamydial inclusions was analyzed by two-photon microscopy. This enabled real-time imaging of chlamydial metabolic changes. (A and B) Color-coded images of τ_2_-NAD(P)H (A) and the corresponding quantitative analysis (B) of chlamydial inclusions. Inside the red dots are chlamydial inclusions. Bars = 10 μm. (C) Numbers of EBs shown by recovery assays at 24 and 36 hpi. (*n *= 51 to 57 from three independent experiments [A and B] and *n* = 3 to 4 [C]) (means ± SEM) (*, *P ≤ *0.05; ***, *P ≤ *0.001; N.S., not significant [by Sidak’s multiple-comparison test]).

Mitochondrial respiration was activated in both productive and β-lactam-induced persistent infection. Thus, we next investigated the chlamydial metabolism that could be linked to mitochondrial activity in productive and persistent infection. Fluorescence lifetime imaging microscopy (FLIM) of NAD(P)H has been demonstrated as a suitable tool for real-time analysis of chlamydial metabolism in living cells (10, 40). Chlamydial metabolism was comparable between productive and penicillin-induced persistent infection at 24 hpi (Fig. 2A and B). In productive infection, however, the fluorescence lifetime of protein-bound NAD(P)H [τ_2_-NAD(P)H] values inside the chlamydial inclusions were reduced at 36 hpi compared to those at 24 hpi (Fig. 2A and B). This finding indicates an increase in metabolically less active EBs at 36 hpi (Fig. 2C), whereas τ_2_-NAD(P)H did not change in aberrant RBs induced by penicillin at between 24 and 36 hpi, suggesting that aberrant RBs remain metabolically active over a longer period (Fig. 2A and B).

### Interconnection of host mitochondrial activity and differentially expressed chlamydial metabolic genes in β-lactam-induced chlamydial persistence.

NAD(P)H detected by FLIM might originate from the oxidative pentose phosphate pathway (PPP) or other energy-rich metabolic pathways, including the TCA cycle, of C. trachomatis (40). However, it is still unclear where NAD(P)H is generated from and how host mitochondrial activity is utilized by β-lactam-induced persistent C. trachomatis. Therefore, we subsequently performed chlamydial transcriptome analysis to identify which chlamydial metabolic pathways are up- or downregulated in penicillin treatment at 24 hpi.

**FIG 3:**
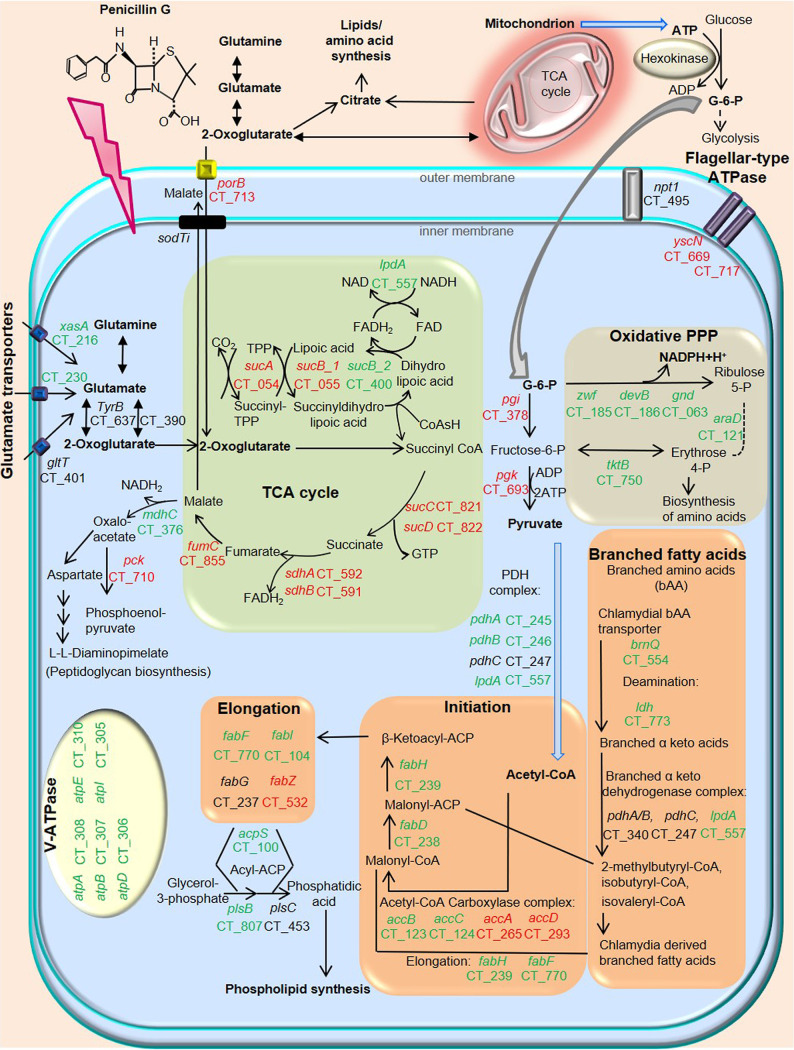
Schematic representation of the metabolic pathways of C. trachomatis during treatment with penicillin. Genes marked in green were significantly upregulated, while genes marked in red were downregulated, in penicillin-induced chlamydial persistence compared to productive infection (*P*_adj_ ≤ 0.05). Chlamydial energy production-associated genes are downregulated in penicillin-induced chlamydial persistence. The chlamydial substrate-specific porin PorB (*porB*), TCA cycle-related genes, and flagellar-type ATPase are downregulated in penicillin-induced chlamydial persistence. Hexokinase of mammalian cells requires mitochondrial ATP to catalyze the conversion of glucose into G-6-P, an essential substrate for chlamydial glycolysis. While chlamydial G-6-P isomerase (*pgi*) is downregulated, the rate-limiting enzyme G-6-P dehydrogenase (*zwf*) in the chlamydial PPP is upregulated in penicillin-induced chlamydial persistence, indicating that G-6-P could be mainly used for the chlamydial PPP. Furthermore, chlamydial fatty acid/phospholipid synthesis as well as acetyl-CoA synthesis are upregulated in penicillin-induced chlamydial persistence. This upregulation is linked to chlamydial phospholipid synthesis. Both acetyl-CoA and oxidative PPP-derived specific NADPH are essential for fatty acid synthesis. FAD, flavin adenine dinucleotide; FADH_2_, reduced flavin adenine dinucleotide; ACP, acyl carrier protein; CoA, coenzyme A.

Chlamydial growth curve and TEM analyses revealed that 24 hpi is the mid-log phase of the RB-to-EB transition in productive infection (Fig. S2A and Fig. S4A). When we analyzed gene expression in penicillin-induced persistence compared to productive C. trachomatis, the substrate-specific porin PorB (*porB*) as well as chlamydial TCA cycle-related genes were downregulated in penicillin-induced persistent infection (Fig. 3 and Table S1). Flagellar-type ATPases were downregulated in penicillin-induced persistent infection (Fig. 3 and Table S1). Chlamydial glucose-6-phosphate (G-6-P) isomerase (*pgi*), which is the key enzyme to catalyze G-6-P to fructose-6-phosphate, was also downregulated in penicillin-induced persistent infection (Fig. 3 and Table S1), whereas the rate-limiting enzyme G-6-P dehydrogenase (*zwf*) in the chlamydial PPP was upregulated in penicillin-induced persistent infection, indicating that G-6-P could be mainly used for the chlamydial oxidative PPP but not for glycolysis in penicillin-induced persistent infection (Fig. 3 and Table S1). In addition, chlamydial acetyl-CoA production and fatty acid synthesis-associated genes, including enoyl-acyl carrier protein reductase (*fabI*) and V-ATPase, were upregulated in penicillin-induced chlamydial persistence (Fig. 3 and Table S1).

10.1128/mBio.00023-21.4 FIG S4 Gene expression of a subset of chlamydial TCA cycle and other metabolic genes in the presence and the absence of penicillin (PEN) or amoxicillin (AMX). (A) One-step growth curve of C. trachomatis. Recoverable C. trachomatis was analyzed at 0, 12, 16, 18, 24, 48, and 72 hpi. C. trachomatis-infected cells were analyzed by qRT-PCR at 18 or 24 hpi. (B) Productive infection versus penicillin-induced persistent infection at 24 hpi. (C) Productive infection versus amoxicillin-induced persistent infection at 24 hpi. (D) Productive infection versus penicillin-induced persistent infection at 18 hpi. Target mRNA expression was normalized to the chlamydial 16S gene (*n *= 3 [A] and *n *= 3 to 16 [B to D]) (means ± SEM) (*, *P ≤ *0.05; **, *P ≤ *0.01; ***, *P ≤ *0.001 [by Student’s *t* test]). Download FIG S4, TIF file, 2.7 MB. Copyright © 2021 Shima et al. 2021 Shima et al. https://creativecommons.org/licenses/by/4.0/ This content is distributed under the terms of the Creative Commons Attribution 4.0 International license.

10.1128/mBio.00023-21.6 TABLE S1 Differentially expressed genes in chlamydial persistence induced by penicillin compared to productive C. trachomatis. Genes with an adjusted *P* value (*P*_adj_) of ≤0.05 are shown. Exp1/Exp2, expression in productive infection/expression in chlamydial persistence. Genes that are not listed are not statistically significant between productive and persistent infection. The functions of plasmid-borne genes are derived from previous reports (64, 65). Download Table S1, XLSX file, 0.07 MB. Copyright © 2021 Shima et al. 2021 Shima et al. https://creativecommons.org/licenses/by/4.0/ This content is distributed under the terms of the Creative Commons Attribution 4.0 International license.

To validate transcriptome analysis in penicillin-induced persistent infection, a subset of chlamydial TCA cycle and other metabolism-related genes were also analyzed by quantitative real-time PCR (qRT-PCR) at 24 hpi. Thus, we could confirm the correlation of gene expressions between RNA sequencing (RNA-seq) and quantitative PCR (qPCR) data (Fig. S4B). We also confirmed a similar trend of expression patterns in a subset of metabolic genes during amoxicillin-induced persistent infection at 24 hpi (Fig. S4C).

**FIG 4:**
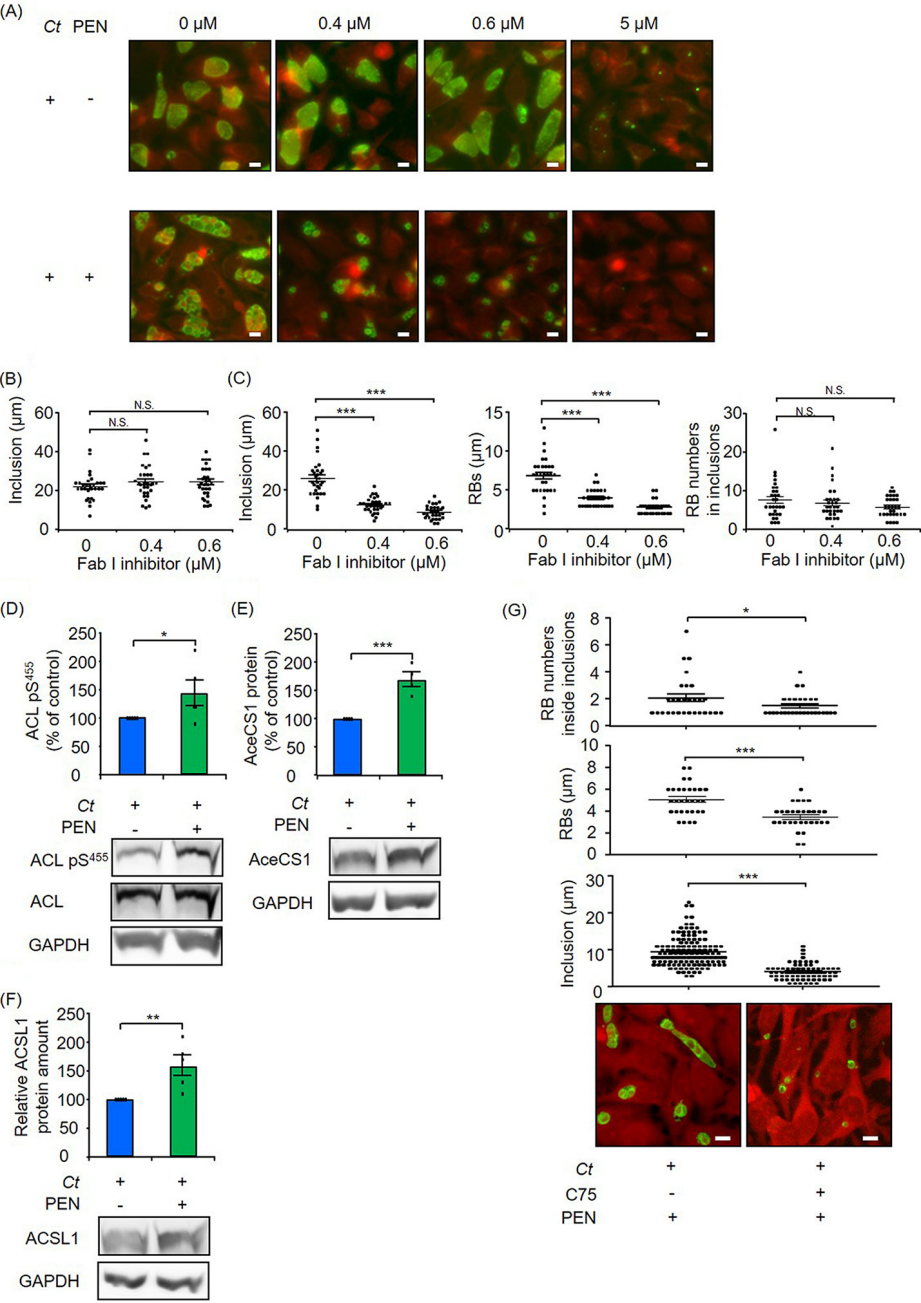
Role of host-pathogen fatty acid synthesis in penicillin (PEN)-induced chlamydial persistence. (A to C) Effect of the bacterial FabI inhibitor. (A) Representative images of productive and penicillin-induced persistent infection in the presence or absence of the FabI inhibitor AFN-1252 at 24 hpi. Green, immunofluorescence staining with FITC-labeled monoclonal chlamydial LOS antibodies; red, Evans blue counterstaining of host cells. Bars = 10 μm. (B) Size of chlamydial inclusions with or without AFN-1252 in productive infection at 24 hpi. (C) Sizes of chlamydial inclusions and aberrant RBs as well as numbers of aberrant RBs inside inclusions with or without AFN-1252 in penicillin-induced chlamydial persistence at 24 hpi. (D to G) Impact of host fatty acid metabolism on penicillin-induced chlamydial persistence. ACL pS^455^ (D) as well as AceCS1 (E) and ACSL1 (F) protein amounts were analyzed by Western blot and densitometric analyses at 24 hpi. ACL pS^455^ was normalized to total ACL, and AceCS1 and ACSL1 protein amounts were normalized to GAPDH. (G) Representative images of penicillin-induced persistent infection in the presence or absence of the host fatty acid synthetic inhibitor C75 at 24 hpi. Green, immunofluorescence staining with FITC-labeled monoclonal chlamydial LOS antibodies; red, Evans blue counterstaining of host cells. Bars = 10 μm. The sizes of chlamydial inclusions and aberrant RBs as well as the numbers of aberrant RBs inside inclusions were analyzed in the presence or absence of C75. *Ct*, C. trachomatis (*n *= 30 from three independent experiments [B and C]; *n *= 5 [D]; *n *= 4 [E]; *n *= 5 [F]; and *n* = 72 to 140 for the size of inclusions, *n *= 31 for the size of aberrant RBs, and *n *= 29 for aberrant RB numbers inside inclusions, from three independent experiments [G]) (means ± SEM) (*, *P ≤ *0.05; **, *P ≤ *0.01; ***, *P ≤ *0.001; N.S., not significant [by Sidak’s multiple-comparison test {B and C} and Student’s *t* test {D to G}]).

In the analysis of RNA-seq, some of the differentially expressed genes in fatty acid biosynthesis were changed <2-fold between productive and penicillin-induced persistent infection. Therefore, we investigated whether these differences are biologically relevant. To examine the correlation between gene expression and chlamydial fatty acid/phospholipid synthesis, we inhibited chlamydial FabI, which is required for the synthesis of fatty acids, influencing the permeability of various molecules and the functions of membrane proteins. While subinhibitory concentrations of 0.4 or 0.6 μM FabI inhibitor (AFN-1252) did not attenuate inclusion formation in productive infection, the same concentrations of the inhibitor significantly reduced the sizes of inclusions and aberrant RBs in penicillin-induced chlamydial persistence, indicating the biological relevance of differentially expressed fatty acid biosynthetic genes in penicillin-induced persistence (Fig. 4A to C).

### Comparison of differentially expressed chlamydial metabolic genes between β-lactam- and IFN-γ-induced chlamydial persistence.

In persistence models, IFN-γ-induced chlamydial persistence is clinically relevant (9, 41–44). In our previous study, we showed that IFN-γ significantly reduced C. trachomatis-induced upregulation of mitochondrial respiration. Thus, this cytokine influences mitochondrial respiration differently than that observed with penicillin-induced persistence (10). Since chlamydial metabolism is strongly influenced by host metabolic activity, we investigated whether the expression pattern of chlamydial metabolic genes also differs in IFN-γ-induced chlamydial persistence.

In line with a previous report (9), we observed persistent morphology and a massive upregulation of the *trpRBA* operon, which was consistent with the deprivation of host l-tryptophan, as well as *euo*, which functions as a regulator of late gene expression in IFN-γ-induced persistent infection compared to productive infection (Table S2 and Fig. S5A) (9). When we analyzed the gene expression patterns of penicillin- and IFN-γ-induced persistent C. trachomatis, only 26% and 38% of up- or downregulated genes were correlated between the two persistent infections, signifying diverse gene expression patterns (Fig. S5B and C). While we showed the upregulation of fatty acid biosynthesis-related genes in β-lactam-induced persistent infection compared to productive infection, this result was not observed in IFN-γ-induced persistent infection (Tables S1 and S2). An interesting similarity is that both inducers cause downregulation of chlamydial TCA cycle-related gene expression (Fig. S5D).

10.1128/mBio.00023-21.5 FIG S5 Comparison of differentially expressed chlamydial genes in penicillin- and IFN-γ-induced C. trachomatis persistence. (A) Inclusion morphology of IFN-γ-induced C. trachomatis persistence was analyzed by TEM. Bar = 10 μm. (B and C) Venn diagram representing the overlap of differentially expressed chlamydial genes in penicillin- and IFN-γ-induced persistence. Upregulated (B) or downregulated (C) chlamydial genes in penicillin- or IFN-γ-induced persistence were compared to productive infection at 24 hpi. (D) Schematic representation of the TCA cycle of C. trachomatis during treatment with IFN-γ. Genes marked in red were downregulated in IFN-γ-induced C. trachomatis persistence compared to productive infection (*P*_adj_ ≤ 0.05). Download FIG S5, TIF file, 2.3 MB. Copyright © 2021 Shima et al. 2021 Shima et al. https://creativecommons.org/licenses/by/4.0/ This content is distributed under the terms of the Creative Commons Attribution 4.0 International license.

10.1128/mBio.00023-21.7 TABLE S2 Differentially expressed genes in chlamydial persistence induced by IFN-γ compared to productive C. trachomatis. Genes with an adjusted *P* value (*P*_adj_) of ≤0.05 are shown. Exp1/Exp2, expression in productive infection/expression in chlamydial persistence. Genes that are not listed are not statistically significant between productive and persistent infection. The functions of plasmid-borne genes are derived from previous reports (64, 65). Download Table S2, XLSX file, 0.04 MB. Copyright © 2021 Shima et al. 2021 Shima et al. https://creativecommons.org/licenses/by/4.0/ This content is distributed under the terms of the Creative Commons Attribution 4.0 International license.

### Impact of host cell fatty acid metabolism on penicillin-induced chlamydial persistence.

Since continued mitochondrial respiration is a unique response in β-lactam-induced persistent infection, we further investigated the role of mitochondria in β-lactam-induced persistent infection. Citrate can be exported from the mitochondrial TCA cycle to the cytosol and becomes a substrate for fatty acid synthesis (25, 45). We showed a higher concentration of citrate in penicillin-induced persistent infection, in accordance with increased host mitochondrial respiration (Fig. 1A and F). Therefore, we determined whether ATP-citrate lyase (ACL), the primary enzyme responsible for the synthesis of acetyl-CoA from citrate for fatty acid synthesis, is activated during penicillin treatment.

Although the protein amount of ACL was not altered, higher catabolic activity of ACL shown by the level of ACL pS^455^ was observed in penicillin-treated cultures at 24 hpi (Fig. 4D). Significant increases in other fatty acid metabolism-associated enzymes such as cytoplasmic acetyl-CoA synthase (AceCS1) and mammalian long-chain acyl-CoA synthetase (ACSL1) were detected during penicillin treatment (Fig. 4E and F). Since higher catabolic activity of ACL was observed in penicillin treatment, we investigated the impact of host fatty acid synthesis on the size of aberrant RBs and inclusion formation in penicillin-induced chlamydial persistence. The fatty acid synthase inhibitor C75 significantly attenuated the sizes of aberrant RBs and inclusions in penicillin-induced chlamydial persistence (Fig. 4G). Furthermore, this inhibitor reduced the number of aberrant RBs in each inclusion, indicating a block of the residual division of aberrant RBs. While we cannot totally exclude the possibility that some of the observed effects of C75 could be due to the inhibition of chlamydial fatty acid synthesis, bacterial and human fatty acid synthases share very little sequence homology (46). Therefore, we argue that activated host mitochondria play a key role in ATP production as well as in the supply of citrate to synthesize host fatty acids, which can be utilized in chlamydial persistence during treatment with β-lactam antimicrobials.

## DISCUSSION

Mitochondria play a pivotal role in different cellular processes such as ATP generation, fatty acid synthesis, redox balance, as well as Ca^2+^ homeostasis (47). It is known that various kinds of proteins such as STAT3, mitochondrial transcription factor A (TFAM), mitofusin (MFN), and dynamin-related protein 1 (DRP1) are associated with biogenesis, fusion, and fission of mitochondria (21, 34, 47). In this study, we demonstrated a chlamydial metabolic switch that is linked to PTP-STAT3 pY^705^-regulated mitochondrial activity in β-lactam-induced persistent infection.

Activation of STAT3 is a common host immune response caused by not only various bacteria such as Salmonella enterica serovar Typhimurium, Mycobacterium tuberculosis, and Helicobacter pylori (48–50) but also viruses, including hepatitis B, hepatitis C, herpes, and human immunodeficiency viruses (51). In contrast, here, we demonstrated that C. trachomatis causes inactivation of STAT3, which is observed in limited viral infections such as severe acute respiratory syndrome (SARS) coronavirus and human papillomavirus type 6 or type 11 (51–53). Importantly, similar PTP-STAT3 regulation was also observed in C. trachomatis-infected primary cells (see Fig. S1R to S1T in the supplemental material).

The protein tyrosine kinases activate STAT3, while PTPs play a key role in hydrolyzing the phosphotyrosine of STAT3. In total, 81 PTPs are active in human cells (54), of which 7 PTPs have been shown to inactivate STAT3 (38). In our study, we showed that mitochondrial activity was significantly enhanced by the inactivation of STAT3 in C. trachomatis infection.

In host cells, 2-oxoglutarate is an important intermediate in the mitochondrial TCA cycle, and glutamine is one of the important carbon sources to be catabolized to 2-oxoglutarate (22–24). The first process of glutamine metabolism involves its conversion to glutamate by the mitochondrial matrix-localized phosphate-dependent glutaminase (55). Glutamate is further catabolized to 2-oxoglutarate, which can be used in the mitochondrial TCA cycle. In C. trachomatis infection, glutamine is rapidly utilized via glutaminolysis and the TCA cycle in order to generate glutamate and other metabolites such as 2-oxoglutarate (24). Since citrate synthase, aconitate hydratase, and isocitrate dehydrogenase are lacking in the chlamydial TCA cycle (22, 23), C. trachomatis has to acquire host cell-derived 2-oxoglutarate using the porin PorB or glutamine/glutamate that can be catabolized to 2-oxoglutarate to fuel its own TCA cycle (23, 24, 56). Therefore, we suggest that mitochondrial activation via PTPs-STAT3 plays a key role in the generation of ATP as well as the enhancement of glutamine metabolism to compensate for the truncated chlamydial TCA cycle in productive C. trachomatis infection.

Chlamydial persistence is induced under treatment with β-lactam antimicrobials (3–7). In line with a previous study (7), we observed aberrant RBs during treatment with penicillin and amoxicillin. Importantly, this phenotype can also activate mitochondria concordant with increased PTPs and reduced STAT3 pY^705^. Since β-lactam antimicrobial treatment could not attenuate host mitochondrial activity, we speculated that bacterial metabolic activity is also sustained under β-lactam antimicrobial treatment. In previous studies, we demonstrated that changes in the fluorescence lifetime of τ_2_-NAD(P)H inside the chlamydial inclusion indicate chlamydial metabolic activity (10, 40). NAD(P)H binds to at least 334 known proteins such as lactate dehydrogenase (LDH) and pyruvate dehydrogenase (PDH) (57). This interaction causes different NAD(P)H fluorescence lifetimes, which are linked to diverse physiological functions (40, 58). Although inclusion morphology is altered in productive and penicillin-induced chlamydial persistence, we documented that chlamydial metabolic activities as defined by the fluorescence lifetime of τ_2_-NAD(P)H were similar in both chlamydial forms at the mid-log phase but not the late log phase of the organism’s developmental cycle.

Because NAD(P)H is generated by different metabolic pathways, we analyzed gene expression using transcriptome analysis and qPCR. To perform this assay, we selected a mid-log phase of the RB-to-EB transition at 24 hpi (Fig. S4A) since we assumed that aberrant RBs are induced from RBs in the developmental cycle. While we morphologically observed a large number of RBs in productive infection at 24 hpi (Fig. S2A), expression of late genes that function in RB-to-EB differentiation was observed at 24 hpi (9, 59). Therefore, we are aware that the interpretation of the observed changes in gene expression could be influenced by the stage of chlamydial development.

In β-lactam-induced chlamydial persistence, PorB (*porB*) and chlamydial TCA cycle-related genes were downregulated compared to productive C. trachomatis at 24 hpi. This downregulation was already observed at 18 hpi before the RB-to-EB transition (Fig. S4A and D). However, the transcriptome analysis revealed that the expression levels of *npt1* (Ct_495) encoding the chlamydial ATP/ADP translocase are similar between productive and penicillin-induced chlamydial persistence (log_2_ fold change of −0.2; adjusted *P* value [*P*_adj_] of 0.06), and V-ATPases were upregulated in penicillin-induced chlamydial persistence. Although it remains to be experimentally proven whether V-ATPase functions as an ATP synthase, these gene expressions might be involved in the acquisition and production of ATP in penicillin-induced chlamydial persistence (22, 60).

Interestingly, fatty acid synthesis as well as oxidative PPP and acetyl-CoA synthesis were upregulated in β-lactam-induced chlamydial persistence compared to productive C. trachomatis. This activation of metabolic pathways is in line with the mechanism of fatty acid synthesis in mammalian cells. NADPH derived from the oxidative PPP as well as acetyl-CoA is essential for fatty acid synthesis (61). As we hypothesized that fatty acid synthesis plays a key role in chlamydial persistence, we could show that the expression of FabI had a stronger impact on chlamydial persistence than productive C. trachomatis. Since the FabI inhibitor does not induce persistence, we suggest that blocking fatty acid synthesis could be an effective treatment for general chlamydial infection.

Since C. trachomatis lacks some genes for fatty acid synthesis, it has to hijack host-derived fatty acids and lipids for chlamydial membrane biogenesis (26–28). In host cells, citrate is exported from the mitochondrial TCA cycle to the cytosol and becomes a substrate for host fatty acid synthesis (25), which is catalyzed by ATP-dependent ACL. Furthermore, reductive glutamine metabolism involves the conversion of 2-oxoglutarate to citrate, which is used as a carbon source to fuel fatty acid synthesis (62). In fact, we observed a higher concentration of citrate and increased catabolic activity of ACL shown by the level of ACL pS^455^ in penicillin treatment at 24 hpi. Besides, we confirmed the higher protein levels of host AceCS1 and ACSL1, which function to produce acetyl-CoA in penicillin-treated cells.

While β-lactam antimicrobials cause dysregulation of peptidoglycan organization and cell division (18–20, 63), IFN-γ depletes tryptophan via the induction of the enzyme indoleamine 2,3-dioxygenase, resulting in the formation of aberrant RBs (41–44). Transcriptome analysis revealed very little overlap of differentially expressed genes between β-lactam- and IFN-γ-induced persistence. While 632 genes were differentially expressed in penicillin-induced persistence, only 357 genes were up- or downregulated in IFN-γ-induced persistence (Fig. S5B and C). Since a large number of differentially expressed transcription-associated genes were also detected in penicillin-induced persistent infection and IFN-γ-induced persistence, this finding highlights the differences in gene expression in the two persistence models.

For reactivation assays, reports show that IFN-γ-induced chlamydial aberrant RBs were reversible and that nearly 100% of aberrant RBs were reactivated after the removal of IFN-γ (9, 17). On the other hand, our results show that the levels of reactivated C. trachomatis in penicillin- and amoxicillin-induced persistence were approximately 3 logs and 1 log lower than in the untreated control, respectively. In line with a previous study (9), our transcriptome analysis revealed that the expression levels of cell division-associated genes, including CT_739, are similar between productive infection and IFN-γ-induced persistence (log_2_ fold change of −0.19; *P*_adj_ of 0.7). However, this expression was significantly suppressed in penicillin-induced persistence (Table S1). Therefore, we suggest that stress induced by β-lactams exerts a stronger impact on reactivation than IFN-γ.

Finally, plasmids are known as a chlamydial virulence factor and impact persistence *in vivo* in a mouse model (64–67). Since we observed that plasmid replication- and maintenance-associated genes were significantly upregulated in both penicillin- and IFN-γ-induced persistence compared to productive infection, their functional relevance and impact on mitochondrial and chlamydial metabolism have to be further elucidated.

**FIG 5:**
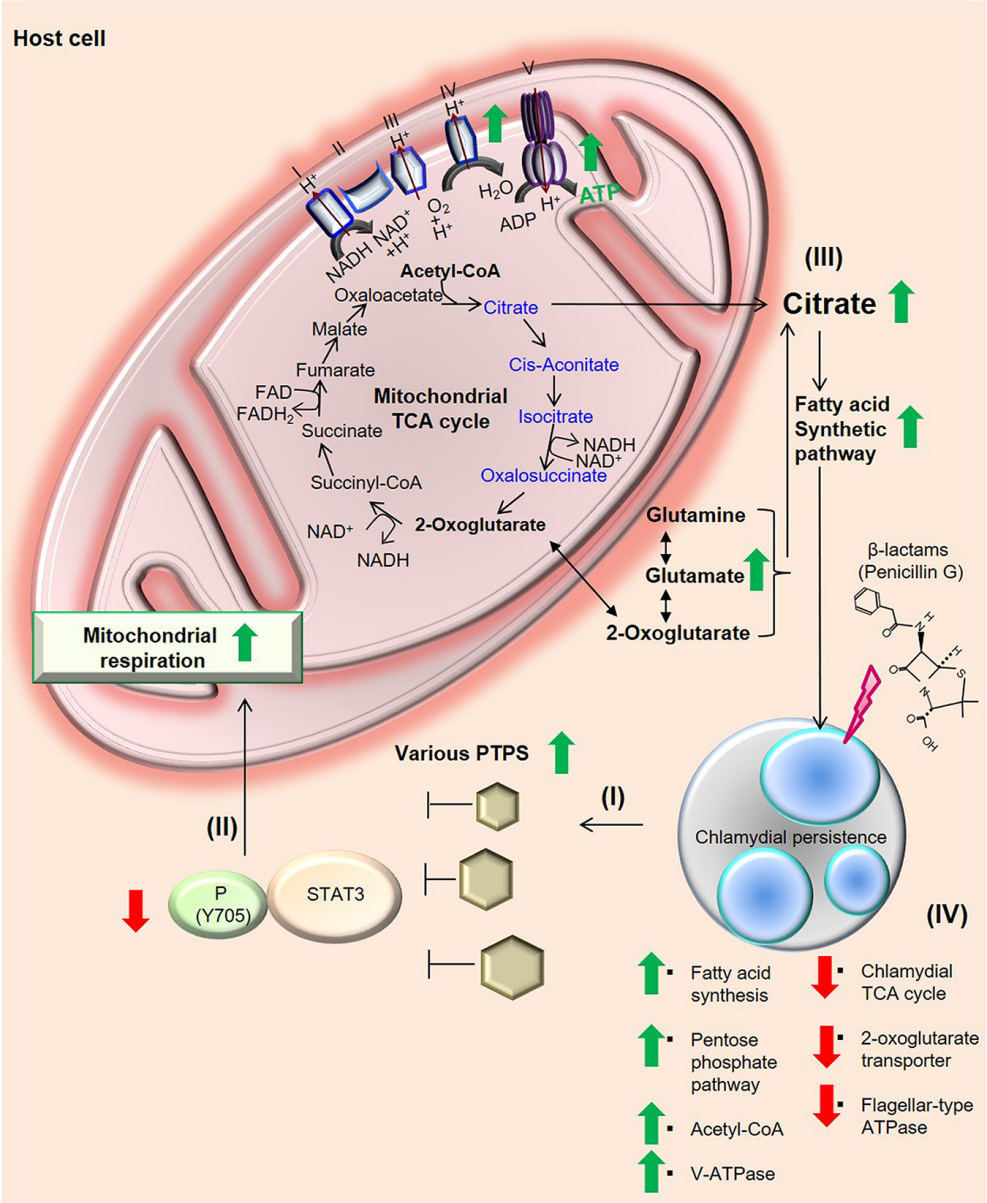
Impact of mitochondrial activity on chlamydial metabolism in β-lactam-induced persistent infection. (I) β-Lactam-induced persistent C. trachomatis inactivates STAT3 by increased host PTPs in epithelial cells. (II) Reduced STAT3 activity functions to accelerate mitochondrial respiration. (III) Activated mitochondria and glutamine metabolism can enhance the production of citrate that can be utilized for host and chlamydial fatty acid/phospholipid synthesis in penicillin-induced chlamydial persistence. (IV) Chlamydial metabolism switched from the tricarboxylic acid cycle to fatty acid synthesis in β-lactam-induced persistent infection. Green arrows indicate upregulation, and red arrows indicate downregulation. Metabolites denoted in blue in the mitochondrial TCA cycle cannot be synthesized in the chlamydial TCA cycle because C. trachomatis lacks metabolic genes encoding citrate synthase, aconitate hydratase, and isocitrate dehydrogenase.

In conclusion, the regulation of PTPs-STAT3 and ACL activity leads to an enhancement of the mitochondrion-fatty acid synthetic axis; thereby, C. trachomatis can undergo metabolic reprogramming to persist in the presence of β-lactam antimicrobials (Fig. 5).

## MATERIALS AND METHODS

### Chemicals and antibodies.

Chemicals were purchased from either Sigma-Aldrich (Deisenhofen, Germany), New England BioLabs GmbH (Frankfurt am Main, Germany), or MedKoo Biosciences, Inc. (Morrisville, NC). Rabbit anti-glyceraldehyde-3-phosphate dehydrogenase (GAPDH), rabbit anti-phospho-Stat3 (Y^705^), rabbit anti-phospho-Stat3 (S^727^), rabbit anti-SOCS3, rabbit anti-ACL, rabbit anti-phospho-ACL (S^455^), rabbit anti-ACSL1, rabbit anti-AceCS1, horseradish peroxidase (HRP)-horse anti-mouse IgG, and HRP-goat anti-rabbit IgG were purchased from Cell Signaling Technology (Frankfurt am Main, Germany). A total oxidative phosphorylation (OXPHOS) rodent Western blot (WB) antibody cocktail was purchased from Abcam (Cambridge, UK). Rabbit anti-STAT3 was purchased from Proteintech Group (Chicago, IL). Two different Stealth RNAi siRNAs for STAT3 (STAT3HSS186130 and STAT3HSS186131) were purchased from Invitrogen (Karlsruhe, Germany).

### Bacterial strains and host cells.

Since the prevalence of urogenital C. trachomatis is caused by serovars D to F, we performed our experiments using C. trachomatis serovar D (ATCC VR885) in this study. HeLa cells (ATCC CCL-2) and primary fibroblasts isolated from C57BL/6J mice (kindly provided from Saleh M. Ibrahim, Lübeck Institute of Experimental Dermatology, University of Lübeck) were used as host cells.

### Cell culture.

All experiments except for the experiment using primary fibroblasts were performed with RPMI 1640 medium supplemented with 5% fetal bovine serum (FBS) (Invitrogen), nonessential amino acids (Gibco/Thermo Fisher Scientific, Waltham, MA), and 2 mM glutamine (Lonza, Walkersville, MD). Dulbecco’s modified Eagle’s medium (DMEM) containing 10% FBS and 2 mM glutamine was used for the culture of primary fibroblast cells. Penicillin G (1 U/ml) or a therapeutically relevant serum concentration of amoxicillin (11 μg/ml) (7) (Sigma-Aldrich) was added at the time of infection to induce chlamydial persistence. For IFN-γ-induced persistence, 50 U/ml IFN-γ was added to the cells 24 h prior to infection.

### XF Cell Mito stress test.

A total of 1.5 × 10^4^ HeLa cells were infected with C. trachomatis (0.7 inclusion-forming units [IFU]/cell) in 24-well XF plates (Seahorse Bioscience, Copenhagen, Denmark). The cells were centrifuged at 700 × *g* for 1 h at 35°C and incubated for 24 h. Afterwards, the Mito stress test kit (Seahorse Bioscience) was used according to the manufacturer’s instructions with the chemicals oligomycin (0.5 μM), carbonyl cyanide-4-(trifluoromethoxy)phenylhydrazone (FCCP) (0.2 μM), and antimycin A (1 μM) plus rotenone (1 μM). In the preliminary experiment, we confirmed that the oligomycin concentration that was used for the XF Cell Mito stress assay had no effect on chlamydial progeny. After the assay, cells from productive infection and chlamydial persistence were fixed with methanol for 10 min at −20°C. Cells were then incubated for 20 min with fluorescein isothiocyanate (FITC)-labeled monoclonal chlamydial lipooligosaccharide (LOS) antibodies (Dako, Glostrup, Denmark). Evans blue counterstaining of host cells was used for better characterization of intracellular inclusions. Staining was confirmed by using a Keyence (Osaka, Japan) BZ-9000 instrument.

### Western blot analysis.

To determine the amount of STAT3 pY^705^, 0.2 × 10^5^ to 5.3 × 10^5^ HeLa or primary fibroblast cells were seeded in 6- or 96-well plates (Greiner Bio-One, Frickenhausen, Germany) and infected with C. trachomatis (0.7 IFU/cell). C. trachomatis-infected cells were lysed with 8 M urea supplemented with 325 U/ml Benzonase nuclease (Sigma-Aldrich) at the indicated times. Cell lysates were diluted into Laemmli buffer (50 mM Tris-HCl [pH 6.8], 2% SDS, 1% 2-mercaptoethanol, 10% glycerol, 0.1% bromophenol blue). On the other hand, we used lysis buffer with bromophenol blue (125 mM Tris-HCl [pH 7.8], 4% SDS, 0.1 M dithiothreitol [DTT], 20% glycerol) to check STAT3 knockdown efficacy. Samples were analyzed by Western blot analysis (Bio-Rad, Hercules, CA) and visualized using enhanced chemiluminescence (ECL) reagent (Millipore and Thermo Fisher Scientific). Images were acquired by using the Fusion FX7 system (Vilber Lourmat, Eberhardzell, Germany), and the density of each band was measured by using Bio-1D software (Vilber Lourmat).

### Electron microscopy.

A total of 5.3 × 10^5^ cells were infected with C. trachomatis (0.7 IFU/cell). C. trachomatis-infected cells were fixed with 2% paraformaldehyde and 2.5% glutaraldehyde in 0.1 M cacodylate buffer for 1 h. Postfixation was performed with 1% OsO_4_ in 0.1 M cacodylate buffer for 2 h. Samples were dehydrated with a graded ethanol series and embedded in Araldite (Fluka, Buchs, Switzerland). Ultrathin sections were stained with uranyl acetate and lead citrate and examined with a JEOL 1011 transmission electron microscope (JEOL, Tokyo, Japan).

### Recovery and reactivation assays of β-lactam-induced persistent C. trachomatis infection.

A total of 1 × 10^5^ HeLa cells were infected with 0.7 IFU/cell of C. trachomatis. C. trachomatis-infected cells were treated with or without 1 U/ml penicillin or 11 μg/ml amoxicillin at the time of infection to induce chlamydial persistence (see Fig. S2D in the supplemental material). The plate was further centrifuged at 700 × *g* for 1 h at 35°C and incubated for 24 h. For reactivation of persistent C. trachomatis, medium was changed to fresh medium in the absence of antimicrobials, and cells were incubated for another 48 h. Afterwards, C. trachomatis-infected cells were harvested, disrupted with glass beads, and inoculated onto 3 × 10^5^ HEp-2 cells (ATCC CCL-23). C. trachomatis infection was visualized by immunofluorescence staining with a mouse anti-chlamydial LOS antibody (kindly provided by Helmut Brade, Borstel, Germany). Chlamydial inclusions were visualized with a Keyence (Osaka, Japan) BZ9000 instrument.

### RNA interference.

Transfection was performed according to the manufacturer’s guidelines (Invitrogen), and STAT3 was transiently silenced by transfection of specific siRNA.

In brief, 2.5 × 10^4^ HeLa cells were seeded into 96-well plates. A mixture of 5 μl Opti-MEM (Invitrogen), 0.2 μl Lipofectamine 2000 (Invitrogen), and 30 nM siRNA was added to cells. The same amount of the negative-control low-GC-content duplex (Invitrogen) was used for the control. After 4 h of incubation, 150 μl RPMI 1640 medium including 5% FBS was added.

### Infection rate and recovery assays in STAT3 knockdown cells.

A total of 5.3 × 10^5^ HeLa cells were infected with C. trachomatis (0.7 IFU/cell) at posttransfection. The plate was further centrifuged at 700 × *g* for 1 h at 35°C and incubated at 37°C under a 5% CO_2_ atmosphere. Afterwards, C. trachomatis infection was analyzed by immunofluorescence staining with mouse anti-chlamydial LOS antibody (green), which was used to visualize chlamydial inclusions (BZ9000; Keyence, Osaka, Japan). Evans blue counterstaining of host cells (red) was used for counting cell numbers. The numbers of recoverable C. trachomatis cells in STAT3 knockdown cells were normalized to those of siRNA control cells.

### Two-photon microscopy.

NAD(P)H autofluorescence was imaged with the DermaInspect two-photon laser scanning microscope (Jen-Lab GmbH, Jena, Germany) (40). In brief, cells were infected with C. trachomatis (0.7 IFU/cell) on cover glass in 50-mm culture dishes with or without penicillin. The cover glass was transferred into a MiniCem chamber before the assay. In each cell, the fluorescence lifetime of protein-bound NADP(H) [τ_2_-NAD(P)H] was measured at 24 and 36 hpi. A 40×/1.3 Plan-Apochromat oil immersion objective was used for visualization. An excitation source at 730 nm was used to measure the fluorescence lifetime of NAD(P)H. The fluorescence lifetime was detected over a period of 49.7 s per image using a time-correlated single-photon counting (TCSPC) system. Afterwards, the data were analyzed by using SPCImage software version 2.9.5.2996 (Becker & Hickl GmbH, Berlin, Germany). In every picture, three inclusions were analyzed from 10 visual fields per chamber in three independent measurements. A region of interest (ROI) was selected inside the whole chlamydial inclusions. Mean values of τ_2_-NAD(P)H of all pixels inside the ROI were calculated.

### NMR analysis.

HeLa cells were infected with C. trachomatis (0.7 IFU/cell) in 6-well plates and cultured in RPMI 1640 medium supplemented with 5% fetal bovine serum with or without 1 U/ml penicillin. The plate was incubated for 24 h at 37°C under a 5% CO_2_ atmosphere. Afterwards, the cells were washed with phosphate-buffered saline (PBS) twice and harvested. For each sample, 5 × 10^6^ cells were used for NMR analysis. Cell extracts were dissolved in pH 7 phosphate buffer with 0.5 mM deuterated TMSP [3-(trimethylsilyl)-2,2,3,3-tetradeuteropropionic acid] as a chemical shift and concentration reference, as described previously (68). Samples were analyzed using a Bruker Avance III 600-MHz nuclear magnetic resonance spectrometer with a room-temperature HCN probe (Bruker). One-dimensional (1D) spectra were acquired using a 1D nuclear Overhauser effect spectroscopy (1D-NOESY) pulse sequence with water presaturation (noesygppr1d), using 1,024 scans, a recycle delay of 4 s, a sweep width of 12 ppm, and 32,768 data points for acquisition. Metabolite concentrations were measured using Chenomx software (version 8.5) and MetaboLab/NMRLab (69, 70). In addition, reference spectra of 2-oxoglutarate, glutamine, glutamate, as well as citrate were acquired in the same buffer.

### Transcriptome analysis.

A total of 2.5 × 10^5^ cells were seeded into 6-well plates and infected with C. trachomatis. Total RNA isolation was performed with the NucleoSpin RNA kit (Macherey-Nagel GmbH & Co. KG, Düren, Germany). Human rRNA was depleted from each sample using Ribo-Zero rRNA removal (human/mouse/rat) kits (Epicentre, Chicago, IL). Afterwards, the obtained human rRNA-depleted RNA was purified with the NucleoSpin RNA kit (Macherey-Nagel) according to the RNA purification protocol. rRNA depletion was checked by Bioanalyzer RNA6000 Pico chip analyses (Agilent Technologies). Depleted RNA preparations were quantified by fluorometric Qubit RNA assays (Life Technologies).

Syntheses of cDNA libraries were performed according to the manufacturer’s protocol (TruSeq RNA sample prep kit; Illumina, Inc.), with minor modifications. Briefly, 45 to 100 ng of human rRNA-depleted RNA was used for cDNA synthesis, followed by adapter ligation and PCR amplification. The resulting cDNA libraries were validated by Agilent DNA 1000 chip analysis (Agilent Technologies), quantified by fluorometric measurement (Qubit high-sensitivity double-stranded DNA [dsDNA HS] assay kit; Life Technologies), and adjusted to 10 nM.

Clonal amplification of cDNA on Illumina v3 flow cells was done using the appropriate cBot recipe (version 8) at a final library concentration of 10 pM. Sequencing was carried out on a HiSeq2500 system according to the manufacturer’s protocol (HiSeq2500 system user guide; Illumina, Inc.) using TruSeq SBS v3 chemistry (Illumina, Inc.). The resulting 100-bp sequence reads were converted to fastq format by using CASAVA 1.8.2.

The sequenced cDNA reads of each sample were analyzed via Babraham Bioinformatics FastQC, a quality control tool for high-throughput sequence data (version 0.11.4; https://www.bioinformatics.babraham.ac.uk/projects/fastqc/). Quality trimming and filtering as well as length filtering were done with PRINSEQ-lite (version 0.20.4). The remaining high-quality sequence reads were mapped against the human reference genome (Genome Reference Consortium human build 38 patch release 2 [GRCh38.p2] [accession number GCF_000001405.28]) and the C. trachomatis D/UW-3/CX reference genome (accession number GCF_000008725.1) combined with the sequence of C. trachomatis D/SotonD1 plasmid pSotonD1 (NCBI reference sequence accession number NC_020986.1) using BWA (version 0.7.17) (71). The mapped reads per *Chlamydia* gene were extracted with featureCounts (version 1.6.0) (72). Normalization and differential expression analysis were done with DESeq2 (71), and as a threshold for differentially expressed genes, an adjusted *P* value of ≤0.05 was used.

### qPCR for analysis of chlamydial gene expression.

A total of 2.5 × 10^5^ cells were seeded in 6-well plates and cultured for 24 h or 18 h. Extraction of total RNA and reverse transcription to cDNA were performed. A LightCycler detection system (Roche Molecular Biochemicals, Mannheim, Germany) was used to perform qPCR. The relative quantification of chlamydial target mRNA expression levels was normalized by chlamydial 16S using the threshold cycle (2^ΔΔ^*^CT^*) method. The primer sequences are listed in Table S3. Statistics were performed with paired Student’s *t* test.

10.1128/mBio.00023-21.8 TABLE S3 Primers used in this study. Download Table S3, XLSX file, 0.01 MB. Copyright © 2021 Shima et al. 2021 Shima et al. https://creativecommons.org/licenses/by/4.0/ This content is distributed under the terms of the Creative Commons Attribution 4.0 International license.

### Expression of PTPs.

A total of 0.3 × 10^5^ to 5.3 × 10^5^ HeLa or primary fibroblast cells were seeded in 6- or 96-well plates. Cells were infected with C. trachomatis (0.7 IFU/cell). PTP expression levels were determined by qPCR as described above. PTP expression levels were normalized by the endogenous control β-actin gene. Primers for PTPN2, PTPN9, PTPN11, PTPN6, PTPRK, PTPRD, and PTPRT were purchased from Bio-Rad Laboratories GmbH (Feldkirchen, Germany).

### Effect of the PTP inhibitor on the phosphorylation of STAT3.

A total of 5.3 × 10^5^ cells were seeded in 6-well plates and cultured for 24 h. Cells were infected with C. trachomatis (0.7 IFU/cell) and centrifuged at 700 × *g* for 1 h at 35°C. Infected cells were incubated at 37°C under a 5% CO_2_ atmosphere for 24 h. Afterwards, they were treated with or without 25 μM sodium orthovanadate (New England BioLabs GmbH) for 3 h.

### Effect of chlamydial FabI and host fatty acid synthetic inhibitors.

A total of 1 × 10^5^ cells were infected with C. trachomatis (0.7 IFU/cell) in the presence or absence of AFN-1252 (MedKoo Biosciences, Inc.). The plate was centrifuged at 700 × *g* for 1 h at 35°C and incubated at 37°C under a 5% CO_2_ atmosphere. The host fatty acid synthetic inhibitor C75 (20 μg/ml) (Sigma-Aldrich) was added at 12 hpi and further incubated for 12 h. Afterwards, C. trachomatis-infected cells were used for immunofluorescence staining with FITC-labeled monoclonal chlamydial LOS antibodies (Dako). A Keyence BZ9000 instrument was used to measure the diameter of chlamydial inclusions.

### Determination of protein concentrations.

A micro-bicinchoninic acid (BCA) protein assay kit (Thermo Fisher Scientific) was used according to the manufacturer’s instructions. In brief, cells were harvested and washed twice in PBS. After centrifugation for 5 min at 300 × *g*, the cell pellet was resuspended in lysis buffer (20 mM HEPES, 150 mM NaCl, 1% Triton X-100, 10% glycerol, protein inhibitor cocktail) and sonicated 3 times for 5 s. After sonication, another centrifugation step followed at 15,000 × *g* for 15 min at 4°C. When the supernatant was collected and used for spectrophotometric analysis to determine the total protein concentration, we confirmed no significant difference in the protein concentrations in C75-treated cells (*n* = 4; mean ± standard error of the mean [SEM]; Sidak’s multiple-comparison test).

### Statistics.

Data are indicated as means ± SEM. Statistical analysis was performed by using GraphPad Prism 7 statistical software. When three or more groups were compared in the experiment, Sidak’s multiple-comparison test was used in cases where one-way analysis of variance showed statistically significant differences (*P* values of ≤0.05). Data between two groups were evaluated using Student’s *t* test. In Sidak’s multiple-comparison and Student’s *t* tests, *P* values of ≤0.05 were considered statistically significant.

### Data availability.

All raw RNA-seq data sets were deposited in the National Center for Biotechnology (NCBI) Sequence Read Archive (SRA) under BioProject accession number PRJNA378340 with SRA accession numbers SRR5834399 and SRR5834397 (productive infection), SRR5834400 and SRR5834398 (penicillin-induced persistence), and SRR13189638 and SRR13189738 (IFN-γ-induced persistence).

## References

[1] Woodhall SC, Gorwitz RJ, Migchelsen SJ, Gottlieb SL, Horner PJ, Geisler WM, Winstanley C, Hufnagel K, Waterboer T, Martin DL, Huston WM, Gaydos CA, Deal C, Unemo M, Dunbar JK, Bernstein K. 2018. Advancing the public health applications of Chlamydia trachomatis serology. Lancet Infect Dis 18:e399–e407. doi:10.1016/S1473-3099(18)30159-2. 29983342 PMC6414067

[2] Omsland A, Sager J, Nair V, Sturdevant DE, Hackstadt T. 2012. Developmental stage-specific metabolic and transcriptional activity of Chlamydia trachomatis in an axenic medium. Proc Natl Acad Sci U S A 109:19781–19785. doi:10.1073/pnas.1212831109. 23129646 PMC3511728

[3] Wyrick PB, Knight ST. 2004. Pre-exposure of infected human endometrial epithelial cells to penicillin in vitro renders Chlamydia trachomatis refractory to azithromycin. J Antimicrob Chemother 54:79–85. doi:10.1093/jac/dkh283. 15163653

[4] Storey C, Chopra I. 2001. Affinities of beta-lactams for penicillin binding proteins of Chlamydia trachomatis and their antichlamydial activities. Antimicrob Agents Chemother 45:303–305. doi:10.1128/AAC.45.1.303-305.2001. 11120983 PMC90278

[5] Lewis ME, Belland RJ, AbdelRahman YM, Beatty WL, Aiyar AA, Zea AH, Greene SJ, Marrero L, Buckner LR, Tate DJ, McGowin CL, Kozlowski PA, O’Brien M, Lillis RA, Martin DH, Quayle AJ. 2014. Morphologic and molecular evaluation of Chlamydia trachomatis growth in human endocervix reveals distinct growth patterns. Front Cell Infect Microbiol 4:71. doi:10.3389/fcimb.2014.00071. 24959423 PMC4050528

[6] Phillips Campbell R, Kintner J, Whittimore J, Schoborg RV. 2012. Chlamydia muridarum enters a viable but non-infectious state in amoxicillin-treated BALB/c mice. Microbes Infect 14:1177–1185. doi:10.1016/j.micinf.2012.07.017. 22943883 PMC3654801

[7] Kintner J, Lajoie D, Hall J, Whittimore J, Schoborg RV. 2014. Commonly prescribed β-lactam antibiotics induce C. trachomatis persistence/stress in culture at physiologically relevant concentrations. Front Cell Infect Microbiol 4:44. doi:10.3389/fcimb.2014.00044. 24783061 PMC3990100

[8] Su H, Caldwell HD. 1995. CD4+ T cells play a significant role in adoptive immunity to Chlamydia trachomatis infection of the mouse genital tract. Infect Immun 63:3302–3308. doi:10.1128/IAI.63.9.3302-3308.1995. 7642259 PMC173455

[9] Belland RJ, Nelson DE, Virok D, Crane DD, Hogan D, Sturdevant D, Beatty WL, Caldwell HD. 2003. Transcriptome analysis of chlamydial growth during IFN-gamma-mediated persistence and reactivation. Proc Natl Acad Sci U S A 100:15971–15976. doi:10.1073/pnas.2535394100. 14673075 PMC307677

[10] Shima K, Kaeding N, Ogunsulire IM, Kaufhold I, Klinger M, Rupp J. 2018. Interferon-γ interferes with host cell metabolism during intracellular Chlamydia trachomatis infection. Cytokine 112:95–101. doi:10.1016/j.cyto.2018.05.039. 29885991

[11] Matsumoto A, Manire GP. 1970. Electron microscopic observations on the effects of penicillin on the morphology of Chlamydia psittaci. J Bacteriol 101:278–285. doi:10.1128/JB.101.1.278-285.1970. 5413965 PMC250478

[12] Clark RB, Schatzki PF, Dalton HP. 1982. Ultrastructural effect of penicillin and cycloheximide on Chlamydia trachomatis strain HAR-13. Med Microbiol Immunol 171:151–159. doi:10.1007/BF02123623. 7162456

[13] Skilton RJ, Cutcliffen LT, Barlow D, Wang Y, Salim O, Lambden PR, Clarke IN. 2009. Penicillin induced persistence in Chlamydia trachomatis: high quality time lapse video analysis of the developmental cycle. PLoS One 4:e7723. doi:10.1371/journal.pone.0007723. 19893744 PMC2769264

[14] Workowski KA, Bolan GA, Centers for Disease Control and Prevention. 2015. Sexually transmitted diseases treatment guidelines, 2015. MMWR Recommend Rep 64:1–137.

[15] World Health Organization. 2016. WHO guidelines for the treatment of Chlamydia trachomatis. World Health Organization, Geneva, Switzerland. 27559553

[16] Hicks LA, Bartoces MG, Roberts RM, Suda KJ, Hunkler RJ, Taylor TH, Jr, Schrag SJ. 2015. US outpatient antibiotic prescribing variation according to geography, patient population, and provider specialty in 2011. Clin Infect Dis 60:1308–1316. doi:10.1093/cid/civ076. 25747410

[17] Reveneau N, Crane DD, Fischer E, Caldwell HD. 2005. Bactericidal activity of first-choice antibiotics against gamma interferon-induced persistent infection of human epithelial cells by Chlamydia trachomatis. Antimicrob Agents Chemother 49:1787–1793. doi:10.1128/AAC.49.5.1787-1793.2005. 15855497 PMC1087634

[18] Liechti GW, Kuru E, Hall E, Kalinda A, Brun YV, VanNieuwenhze M, Maurelli AT. 2014. A new metabolic cell-wall labelling method reveals peptidoglycan in Chlamydia trachomatis. Nature 506:507–510. doi:10.1038/nature12892. 24336210 PMC3997218

[19] Klöckner A, Otten C, Derouaux A, Vollmer W, Bühl H, De Benedetti S, Münch D, Josten M, Mölleken K, Sahl H-G, Henrichfreise B. 2014. AmiA is a penicillin target enzyme with dual activity in the intracellular pathogen Chlamydia pneumoniae. Nat Commun 5:4201. doi:10.1038/ncomms5201. 24953137 PMC4083426

[20] Cox JV, Abdelrahman YM, Ouellette SP. 2020. Penicillin-binding proteins regulate multiple steps in the polarized cell division process of Chlamydia. Sci Rep 10:12588. doi:10.1038/s41598-020-69397-x. 32724139 PMC7387471

[21] Chowdhury SR, Reimer A, Sharan M, Kozjak-Pavlovic V, Eulalio A, Prusty BK, Fraunholz M, Karunakaran K, Rudel T. 2017. Chlamydia preserves the mitochondrial network necessary for replication via microRNA-dependent inhibition of fission. J Cell Biol 216:1071–1089. doi:10.1083/jcb.201608063. 28330939 PMC5379946

[22] Stephens RS, Kalman S, Lammel C, Fan J, Marathe R, Aravind L, Mitchell W, Olinger L, Tatusov RL, Zhao Q, Koonin EV, Davis RW. 1998. Genome sequence of an obligate intracellular pathogen of humans: Chlamydia trachomatis. Science 282:754–759. doi:10.1126/science.282.5389.754. 9784136

[23] Kubo A, Stephens RS. 2001. Substrate-specific diffusion of select dicarboxylates through Chlamydia trachomatis PorB. Microbiology (Reading) 147:3135–3140. doi:10.1099/00221287-147-11-3135. 11700364

[24] Rajeeve K, Vollmuth N, Janaki-Raman S, Wulff TF, Baluapuri A, Dejure FR, Huber C, Fink J, Schmalhofer M, Schmitz W, Sivadasan R, Eilers M, Wolf E, Eisenreich W, Schulze A, Seibel J, Rudel T. 2020. Reprogramming of host glutamine metabolism during Chlamydia trachomatis infection and its key role in peptidoglycan synthesis. Nat Microbiol 5:1390–1402. doi:10.1038/s41564-020-0762-5. 32747796

[25] Zaidi N, Swinnen JV, Smans K. 2012. ATP-citrate lyase: a key player in cancer metabolism. Cancer Res 72:3709–3714. doi:10.1158/0008-5472.CAN-11-4112. 22787121

[26] Yao J, Cherian PT, Frank MW, Rock CO. 2015. Chlamydia trachomatis relies on autonomous phospholipid synthesis for membrane biogenesis. J Biol Chem 290:18874–18888. doi:10.1074/jbc.M115.657148. 25995447 PMC4521007

[27] Yao J, Dodson VJ, Frank MW, Rock CO. 2015. Chlamydia trachomatis scavenges host fatty acids for phospholipid synthesis via an acyl-acyl carrier protein synthetase. J Biol Chem 290:22163–22173. doi:10.1074/jbc.M115.671008. 26195634 PMC4571967

[28] Yao J, Abdelrahman YM, Robertson RM, Cox JV, Belland RJ, White SW, Rock CO. 2014. Type II fatty acid synthesis is essential for the replication of Chlamydia trachomatis. J Biol Chem 289:22365–22376. doi:10.1074/jbc.M114.584185. 24958721 PMC4139244

[29] Van Ooij C, Kalman L, Van Ijzendoorn S, Nishijima M, Hanada K, Mostov K, Engel JN. 2000. Host cell-derived sphingolipids are required for the intracellular growth of Chlamydia trachomatis. Cell Microbiol 2:627–637. doi:10.1046/j.1462-5822.2000.00077.x. 11207614

[30] Elwell CA, Jiang S, Kim JH, Lee A, Wittmann T, Hanada K, Melancon P, Engel JN. 2011. Chlamydia trachomatis co-opts GBF1 and CERT to acquire host sphingomyelin for distinct roles during intracellular development. PLoS Pathog 7:e1002198. doi:10.1371/journal.ppat.1002198. 21909260 PMC3164637

[31] Derré I, Swiss R, Agaisse H. 2011. The lipid transfer protein CERT interacts with the Chlamydia inclusion protein IncD and participates to ER-Chlamydia inclusion membrane contact sites. PLoS Pathog 7:e1002092. doi:10.1371/journal.ppat.1002092. 21731489 PMC3121800

[32] Vromman F, Subtil A. 2014. Exploitation of host lipids by bacteria. Curr Opin Microbiol 17:38–45. doi:10.1016/j.mib.2013.11.003. 24581691

[33] Sala D, Cunningham TJ, Stec MJ, Etxaniz U, Nicoletti C, Dall’Agnese A, Puri PL, Duester G, Latella L, Sacco A. 2019. The Stat3-Fam3a axis promotes muscle stem cell myogenic lineage progression by inducing mitochondrial respiration. Nat Commun 10:1796. doi:10.1038/s41467-019-09746-1. 30996264 PMC6470137

[34] Demaria M, Giorgi C, Lebiedzinska M, Esposito G, D’Angeli L, Bartoli A, Gough DJ, Turkson J, Levy DE, Watson CJ, Wieckowski MR, Provero P, Pinton P, Poli V. 2010. A STAT3-mediated metabolic switch is involved in tumour transformation and STAT3 addiction. Aging (Albany NY) 2:823–842. doi:10.18632/aging.100232. 21084727 PMC3006025

[35] Camporeale A, Demaria M, Monteleone E, Giorgi C, Wieckowski MR, Pinton P, Poli V. 2014. STAT3 activities and energy metabolism: dangerous liaisons. Cancers (Basel) 6:1579–1596. doi:10.3390/cancers6031579. 25089666 PMC4190557

[36] Pensa S, Demaria M, Avalle L, Barbieri I, Camporeale A, Poli V. 2012. From tissue invasion to glucose metabolism: the many aspects of signal transducer and activator of transcription 3 pro-oncogenic activities. Horm Mol Biol Clin Invest 10:217–225. doi:10.1515/hmbci-2012-0006. 25436678

[37] Wegrzyn J, Potla R, Chwae Y-J, Sepuri NBV, Zhang Q, Koeck T, Derecka M, Szczepanek K, Szelag M, Gornicka A, Moh A, Moghaddas S, Chen Q, Bobbili S, Cichy J, Dulak J, Baker DP, Wolfman A, Stuehr D, Hassan MO, Fu X-Y, Avadhani N, Drake JI, Fawcett P, Lesnefsky EJ, Larner AC. 2009. Function of mitochondrial Stat3 in cellular respiration. Science 323:793–797. doi:10.1126/science.1164551. 19131594 PMC2758306

[38] Kim M, Morales LD, Jang I-S, Cho Y-Y, Kim DJ. 2018. Protein tyrosine phosphatases as potential regulators of STAT3 signaling. Int J Mol Sci 19:2708. doi:10.3390/ijms19092708. PMC6164089 30208623

[39] Shuai K, Liu B. 2003. Regulation of JAK-STAT signalling in the immune system. Nat Rev Immunol 3:900–911. doi:10.1038/nri1226. 14668806

[40] Szaszák M, Steven P, Shima K, Orzekowsky-Schröder R, Hüttmann G, König IR, Solbach W, Rupp J. 2011. Fluorescence lifetime imaging unravels C. trachomatis metabolism and its crosstalk with the host cell. PLoS Pathog 7:e1002108. doi:10.1371/journal.ppat.1002108. 21779161 PMC3136453

[41] Beatty WL, Morrison RP, Byrne GI. 1994. Persistent chlamydiae: from cell culture to a paradigm for chlamydial pathogenesis. Microbiol Rev 58:686–699. doi:10.1128/MR.58.4.686-699.1994. 7854252 PMC372987

[42] Schoborg RV. 2011. Chlamydia persistence—a tool to dissect chlamydia-host interactions. Microbes Infect 13:649–662. doi:10.1016/j.micinf.2011.03.004. 21458583 PMC3636554

[43] Beatty WL, Byrne GI, Morrison RP. 1993. Morphologic and antigenic characterization of interferon gamma-mediated persistent Chlamydia trachomatis infection in vitro. Proc Natl Acad Sci U S A 90:3998–4002. doi:10.1073/pnas.90.9.3998. 8387206 PMC46433

[44] Loomis WP, Starnbach MN. 2002. T cell responses to Chlamydia trachomatis. Curr Opin Microbiol 5:87–91. doi:10.1016/s1369-5274(02)00291-6. 11834375

[45] Mashima T, Seimiya H, Tsuruo T. 2009. De novo fatty-acid synthesis and related pathways as molecular targets for cancer therapy. Br J Cancer 100:1369–1372. doi:10.1038/sj.bjc.6605007. 19352381 PMC2694429

[46] Sadowski MC, Pouwer RH, Gunter JH, Lubik AA, Quinn RJ, Nelson CC. 2014. The fatty acid synthase inhibitor triclosan: repurposing an anti-microbial agent for targeting prostate cancer. Oncotarget 5:9362–9381. doi:10.18632/oncotarget.2433. 25313139 PMC4253440

[47] Kiriyama Y, Nochi H. 2018. Intra- and intercellular quality control mechanisms of mitochondria. Cells 7:1. doi:10.3390/cells7010001. PMC5789274 29278362

[48] Lin T, Bost KL. 2004. STAT3 activation in macrophages following infection with Salmonella. Biochem Biophys Res Commun 321:828–834. doi:10.1016/j.bbrc.2004.07.039. 15358102

[49] Zhao J, Dong Y, Kang W, Go MY, Tong JH, Ng EK, Chiu PW, Cheng AS, To KF, Sung JJ, Yu J. 2014. Helicobacter pylori-induced STAT3 activation and signalling network in gastric cancer. Oncoscience 1:468–475. doi:10.18632/oncoscience.62. 25594045 PMC4284628

[50] Queval CJ, Song O-R, Deboosère N, Delorme V, Debrie A-S, Iantomasi R, Veyron-Churlet R, Jouny S, Redhage K, Deloison G, Baulard A, Chamaillard M, Locht C, Brodin P. 2016. STAT3 represses nitric oxide synthesis in human macrophages upon Mycobacterium tuberculosis infection. Sci Rep 6:29297. doi:10.1038/srep29297. 27384401 PMC4935992

[51] Chang Z, Wang Y, Zhou X, Long J-E. 2018. STAT3 roles in viral infection: antiviral or proviral? Future Virol 13:557–574. doi:10.2217/fvl-2018-0033. 32201498 PMC7079998

[52] Mizutani T, Fukushi S, Murakami M, Hirano T, Saijo M, Kurane I, Morikawa S. 2004. Tyrosine dephosphorylation of STAT3 in SARS coronavirus-infected Vero E6 cells. FEBS Lett 577:187–192. doi:10.1016/j.febslet.2004.10.005. 15527783 PMC7125663

[53] Sun S, Steinberg BM. 2002. PTEN is a negative regulator of STAT3 activation in human papillomavirus-infected cells. J Gen Virol 83:1651–1658. doi:10.1099/0022-1317-83-7-1651. 12075083

[54] Xu Y, Fisher GJ. 2012. Receptor type protein tyrosine phosphatases (RPTPs)—roles in signal transduction and human disease. J Cell Commun Signal 6:125–138. doi:10.1007/s12079-012-0171-5. 22851429 PMC3421019

[55] Stumvoll M, Perriello G, Meyer C, Gerich J. 1999. Role of glutamine in human carbohydrate metabolism in kidney and other tissues. Kidney Int 55:778–792. doi:10.1046/j.1523-1755.1999.055003778.x. 10027916

[56] Yang M, Rajeeve K, Rudel T, Dandekar T. 2019. Comprehensive flux modeling of Chlamydia trachomatis proteome and qRT-PCR data indicate biphasic metabolic differences between elementary bodies and reticulate bodies during infection. Front Microbiol 10:2350. doi:10.3389/fmicb.2019.02350. 31681215 PMC6803457

[57] Sharick JT, Favreau PF, Gillette AA, Sdao SM, Merrins MJ, Skala MC. 2018. Protein-bound NAD(P)H lifetime is sensitive to multiple fates of glucose carbon. Sci Rep 8:5456. doi:10.1038/s41598-018-23691-x. 29615678 PMC5883019

[58] Lakowicz JR, Szmacinski H, Nowaczyk K, Johnson ML. 1992. Fluorescence lifetime imaging of free and protein-bound NADH. Proc Natl Acad Sci U S A 89:1271–1275. doi:10.1073/pnas.89.4.1271. 1741380 PMC48431

[59] Chiarelli TJ, Grieshaber NA, Omsland A, Remien CH, Grieshaber SS. 2020. Single-inclusion kinetics of Chlamydia trachomatis development. mSystems 5:e00689-20. doi:10.1128/mSystems.00689-20. 33051378 PMC7567582

[60] Schep DG, Zhao J, Rubinstein JL. 2016. Models for the a subunits of the Thermus thermophilus V/A-ATPase and Saccharomyces cerevisiae V-ATPase enzymes by cryo-EM and evolutionary covariance. Proc Natl Acad Sci U S A 113:3245–3250. doi:10.1073/pnas.1521990113. 26951669 PMC4812769

[61] Patra KC, Hay N. 2014. The pentose phosphate pathway and cancer. Trends Biochem Sci 39:347–354. doi:10.1016/j.tibs.2014.06.005. 25037503 PMC4329227

[62] Fendt S-M, Bell EL, Keibler MA, Olenchock BA, Mayers JR, Wasylenko TM, Vokes NI, Guarente L, Vander Heiden MG, Stephanopoulos G. 2013. Reductive glutamine metabolism is a function of the α-ketoglutarate to citrate ratio in cells. Nat Commun 4:2236. doi:10.1038/ncomms3236. 23900562 PMC3934748

[63] McCoy AJ, Adams NE, Hudson AO, Gilvarg C, Leustek T, Maurelli AT. 2006. l,l-Diaminopimelate aminotransferase, a trans-kingdom enzyme shared by Chlamydia and plants for synthesis of diaminopimelate/lysine. Proc Natl Acad Sci U S A 103:17909–17914. doi:10.1073/pnas.0608643103. 17093042 PMC1693846

[64] Yang C, Kari L, Lei L, Carlson JH, Ma L, Couch CE, Whitmire WM, Bock K, Moore I, Bonner C, McClarty G, Caldwell HD. 2020. Chlamydia trachomatis plasmid gene protein 3 is essential for the establishment of persistent infection and associated immunopathology. mBio 11:e01902-20. doi:10.1128/mBio.01902-20. 32817110 PMC7439461

[65] Zhong G. 2017. Chlamydial plasmid-dependent pathogenicity. Trends Microbiol 25:141–152. doi:10.1016/j.tim.2016.09.006. 27712952 PMC5272858

[66] Li Z, Chen D, Zhong Y, Wang S, Zhong G. 2008. The chlamydial plasmid-encoded protein pgp3 is secreted into the cytosol of Chlamydia-infected cells. Infect Immun 76:3415–3428. doi:10.1128/IAI.01377-07. 18474640 PMC2493211

[67] Liu Y, Huang Y, Yang Z, Sun Y, Gong S, Hou S, Chen C, Li Z, Liu Q, Wu Y, Baseman J, Zhong G. 2014. Plasmid-encoded Pgp3 is a major virulence factor for Chlamydia muridarum to induce hydrosalpinx in mice. Infect Immun 82:5327–5335. doi:10.1128/IAI.02576-14. 25287930 PMC4249284

[68] Tiziani S, Lodi A, Khanim FL, Viant MR, Bunce CM, Günther UL. 2009. Metabolomic profiling of drug responses in acute myeloid leukaemia cell lines. PLoS One 4:e4251. doi:10.1371/journal.pone.0004251. 19158949 PMC2621336

[69] Günther UL, Ludwig C, Rüterjans H. 2000. NMRLAB—advanced NMR data processing in matlab. J Magn Reson 145:201–208. doi:10.1006/jmre.2000.2071. 10910688

[70] Ludwig C, Günther UL. 2011. MetaboLab—advanced NMR data processing and analysis for metabolomics. BMC Bioinformatics 12:366. doi:10.1186/1471-2105-12-366. 21914187 PMC3179975

[71] Love MI, Huber W, Anders S. 2014. Moderated estimation of fold change and dispersion for RNA-seq data with DESeq2. Genome Biol 15:550. doi:10.1186/s13059-014-0550-8. 25516281 PMC4302049

[72] Liao Y, Smyth GK, Shi W. 2014. featureCounts: an efficient general purpose program for assigning sequence reads to genomic features. Bioinformatics 30:923–930. doi:10.1093/bioinformatics/btt656. 24227677

