# Supplementary figures and images for: Regulation of the Mitochondrion-Fatty Acid Axis for the Metabolic Reprogramming of Chlamydia trachomatis during Treatment with β-Lactam Antimicrobials
Source: mBio. 2021 Mar 30;12(2):e00023-21. doi: 10.1128/mBio.00023-21 (PMC8092193; doi:10.1128/mBio.00023-21)

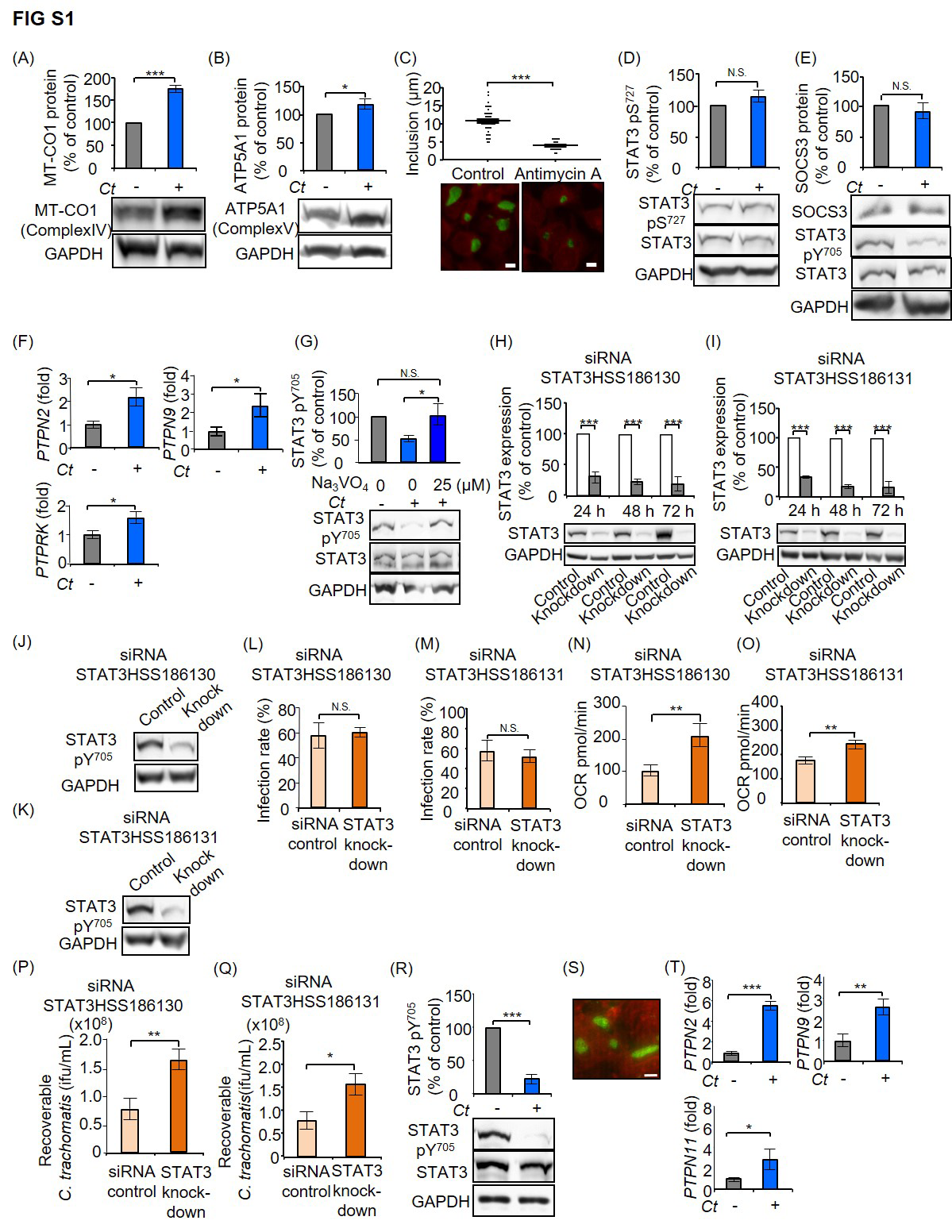

Supplement: FIG S1 [file mBio.00023-21-sf001.tif]

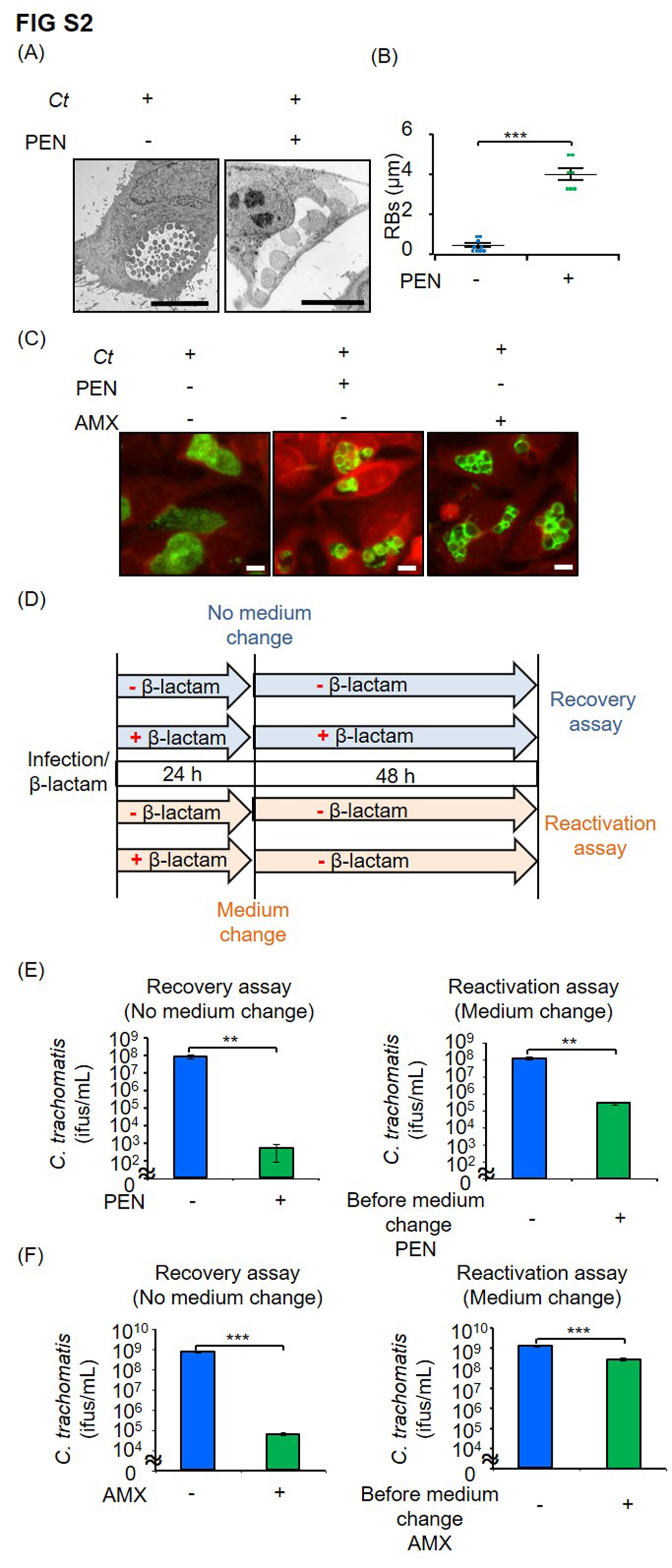

Supplement: FIG S2 [file mBio.00023-21-sf002.tif]

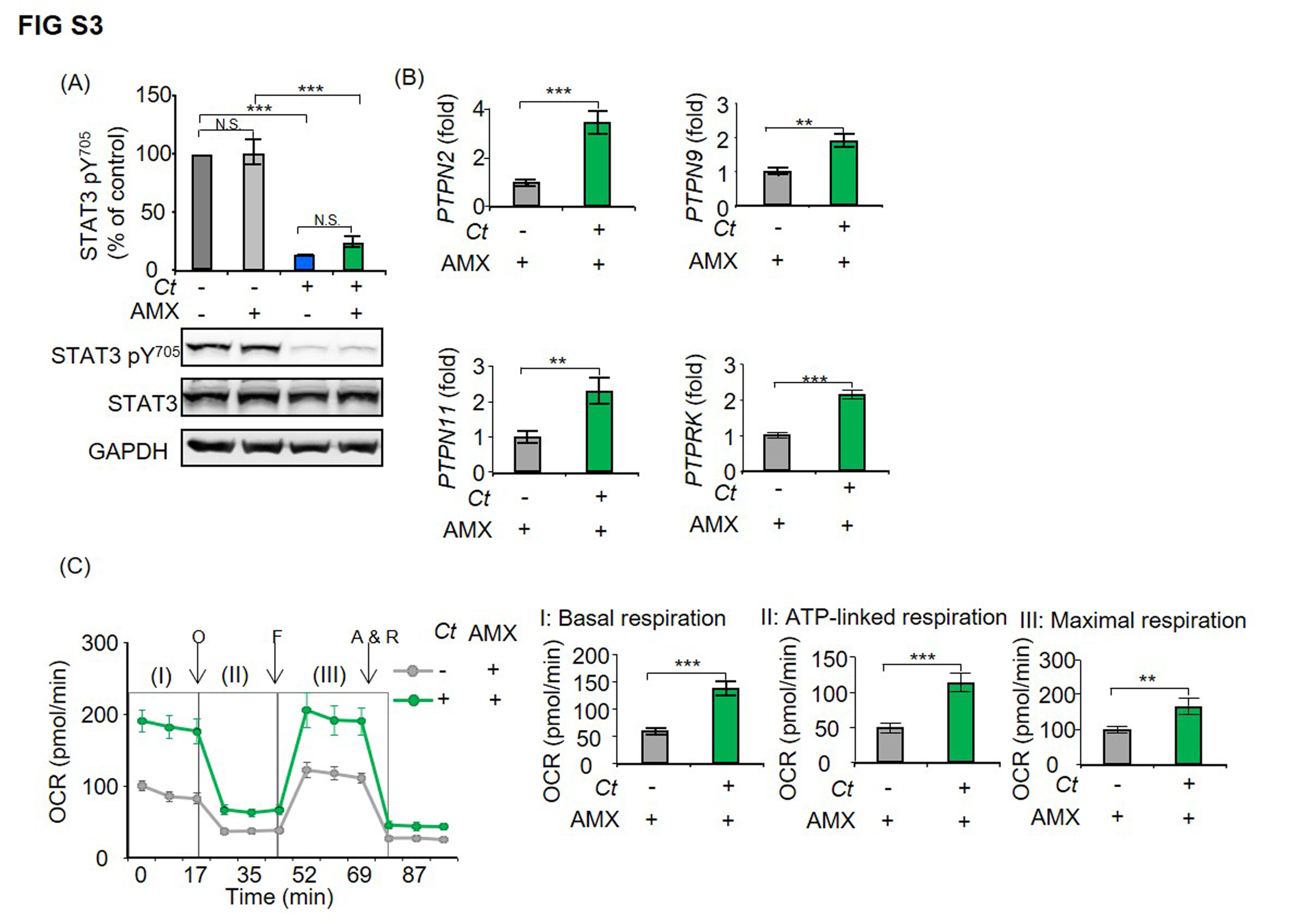

Supplement: FIG S3 [file mBio.00023-21-sf003.tif]

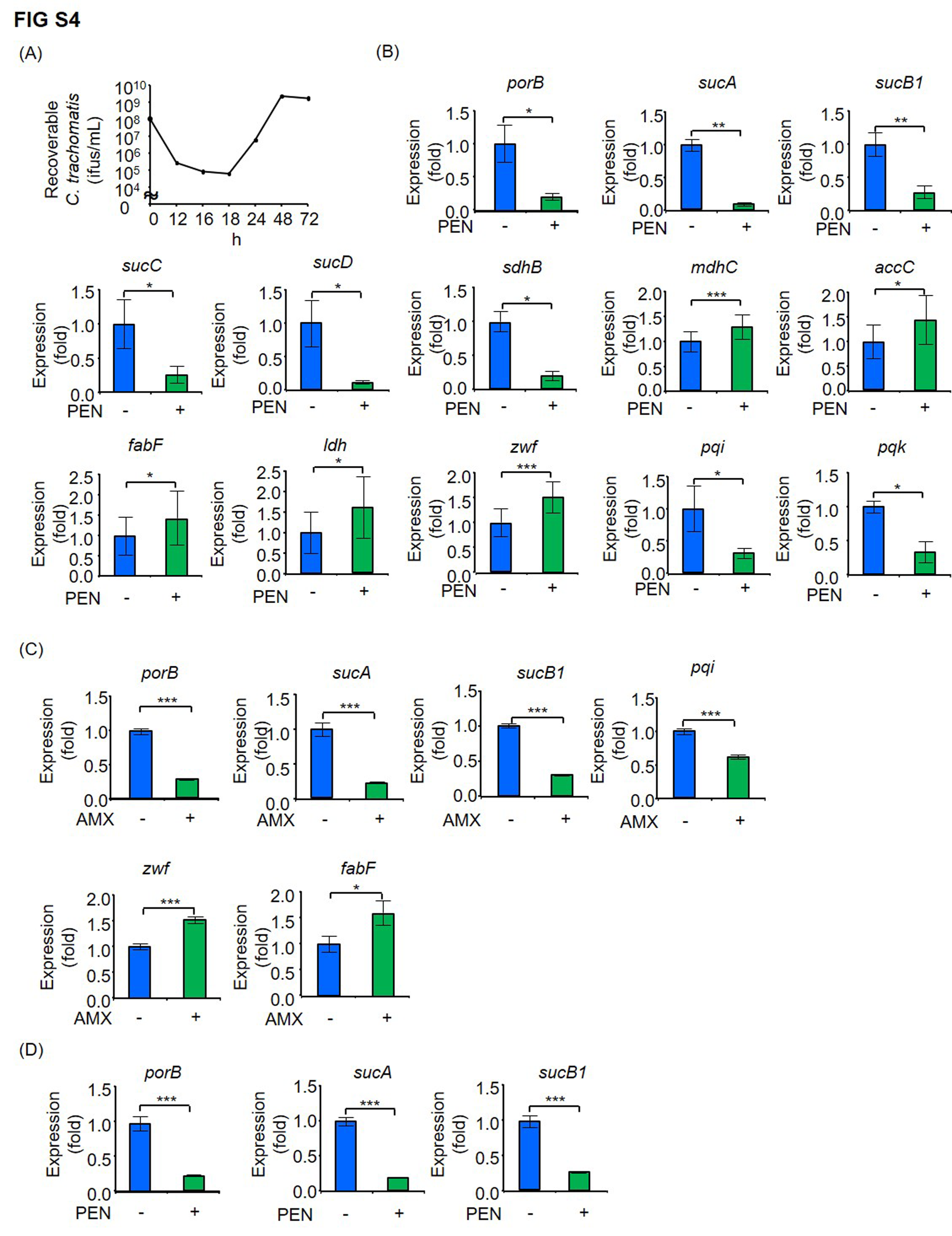

Supplement: FIG S4 [file mBio.00023-21-sf004.tif]

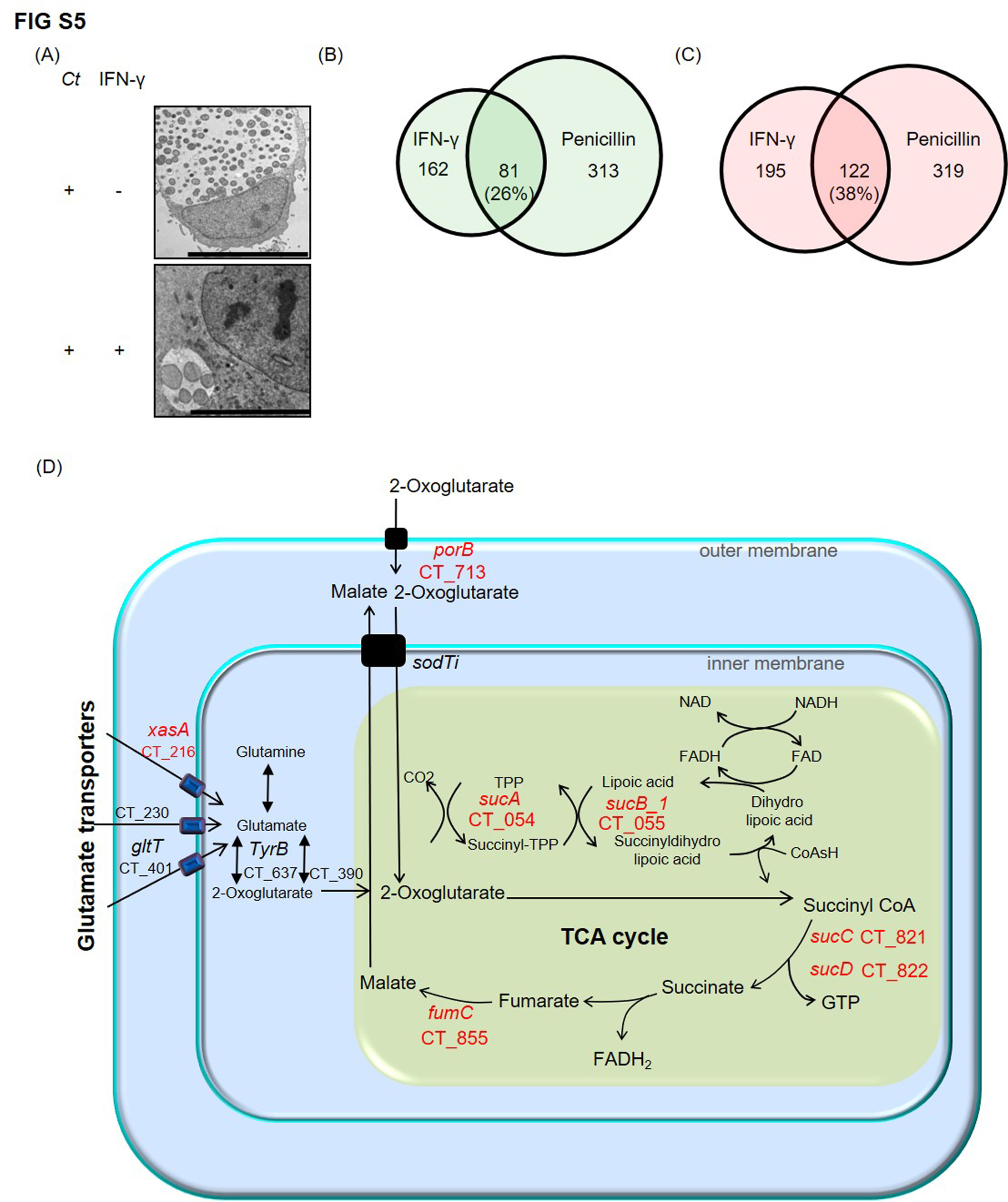

Supplement: FIG S5 [file mBio.00023-21-sf005.tif]
